# Gene content of seawater microbes is a strong predictor of water chemistry across the Great Barrier Reef

**DOI:** 10.1186/s40168-024-01972-0

**Published:** 2025-01-16

**Authors:** Marko Terzin, Steven J. Robbins, Sara C. Bell, Kim-Anh Lê Cao, Renee K. Gruber, Pedro R. Frade, Nicole S. Webster, Yun Kit Yeoh, David G. Bourne, Patrick W. Laffy

**Affiliations:** 1 Australian Institute of Marine Science, PMB no3 Townsville MC, Townsville, QLD 4810 Australia; 2 College of Science and Engineering, James Cook University, Townsville, 4811 Australia; 3 AIMS@JCU, James Cook University, Townsville, QLD 4811 Australia; 4 Australian Centre for Ecogenomics, University of Queensland, St Lucia, Brisbane, QLD 4072 Australia; 5 Melbourne Integrative Genomics and School of Mathematics and Statistics, University of Melbourne, Parkville, Melbourne, VIC 3052 Australia; 6 Natural History Museum Vienna, Vienna, 1010 Austria; 7 Institute for Marine and Antarctic Studies, University of Tasmania, Hobart, TAS 7001 Australia

**Keywords:** Coral reefs, Seawater microbiome, *Synechococcus*, *Prochlorococcus*, Microbial loop, Metagenomics, Environmental monitoring, Microbial indicators, Great Barrier Reef

## Abstract

**Background:**

Seawater microbes (bacteria and archaea) play essential roles in coral reefs by facilitating nutrient cycling, energy transfer, and overall reef ecosystem functioning. However, environmental disturbances such as degraded water quality and marine heatwaves, can impact these vital functions as seawater microbial communities experience notable shifts in composition and function when exposed to stressors. This sensitivity highlights the potential of seawater microbes to be used as indicators of reef health. Microbial indicator analysis has centered around measuring the taxonomic composition of seawater microbial communities, but this can obscure heterogeneity of gene content between taxonomically similar microbes, and thus, microbial functional genes have been hypothesized to have more scope for predictive potential, though empirical validation for this hypothesis is still pending. Using a metagenomics study framework, we establish a functional baseline of seawater microbiomes across offshore Great Barrier Reef (GBR) sites to compare the diagnostic value between taxonomic and functional information in inferring continuous physico-chemical metrics in the surrounding reef.

**Results:**

Integrating gene-centric metagenomics analyses with 17 physico-chemical variables (temperature, salinity, and particulate and dissolved nutrients) across 48 reefs revealed that associations between microbial functions and environmental parameters were twice as stable compared to taxonomy-environment associations. Distinct seasonal variations in surface water chemistry were observed, with nutrient concentrations up to threefold higher during austral summer, explained by enhanced production of particulate organic matter (POM) by photoautotrophic picocyanobacteria, primarily *Synechococcus*. In contrast, nutrient levels were lower in winter, and POM production was also attributed to *Prochlorococcus*. Additionally, heterotrophic microbes (e.g., *Rhodospirillaceae*, *Burkholderiaceae*, *Flavobacteriaceae*, and *Rhodobacteraceae*) were enriched in reefs with elevated dissolved organic carbon (DOC) and phytoplankton-derived POM, encoding functional genes related to membrane transport, sugar utilization, and energy metabolism. These microbes likely contribute to the coral reef microbial loop by capturing and recycling nutrients derived from *Synechococcus* and *Prochlorococcus*, ultimately transferring nutrients from picocyanobacterial primary producers to higher trophic levels.

**Conclusion:**

This study reveals that functional information in reef-associated seawater microbes more robustly associates with physico-chemical variables than taxonomic data, highlighting the importance of incorporating microbial function in reef monitoring initiatives. Our integrative approach to mine for stable seawater microbial biomarkers can be expanded to include additional continuous metrics of reef health (e.g., benthic cover of corals and macroalgae, fish counts/biomass) and may be applicable to other large-scale reef metagenomics datasets beyond the GBR.

Video Abstract

**Supplementary Information:**

The online version contains supplementary material available at 10.1186/s40168-024-01972-0.

## Background

Coral reefs globally are increasingly subjected to the impacts of climate change and anthropogenic activity [1–3], driving declines in the health of these critical ecosystems [4, 5]. Early identification of adverse environmental conditions and declining reef health is important for the development of management strategies that can effectively mitigate the effects of environmental pressures [6–10]. Free-living seawater microorganisms are the first responders to environmental change on reefs owing to their rapid turnover rates measured in hours or days [11–13]. The utility of microbes for reef monitoring has been previously proposed (reviewed in [8, 9, 14, 15]), with many studies documenting rapid changes in the structure of seawater microbial communities on reefs subjected to environmental stress [16–21]. Seawater microbiomes have been shown to be up to fivefold more accurate compared to sediment and host-associated (coral, sponge, and macroalgae) microbiomes in predicting temperature and nutrient concentrations on reefs [11]. This was attributed to planktonic communities being more uniform in their spatial and temporal distribution across reef waters in contrast to sediment microbes, which were highly site specific (i.e., influenced by sediment grain size and chemical composition), and host microbiomes strongly influenced by host genotype [11, 22]. Moreover, seawater can be easily and non-destructively collected alongside in situ reef health surveys; hence, there is realistic scope to complement ongoing reef monitoring programs with seawater microbial observations [10, 15].

Microbial communities in seawater are influenced by various oceanographic processes such as transport, mixing, resuspension, and shelf upwelling, in addition to niches associated with water chemistry and/or interactions with surrounding benthic and pelagic communities. As such, the challenge with using seawater microbial communities as indicators of reef health is in assessing their associations to different environmental factors (e.g., temperature, salinity, nutrient concentrations, and local biodiversity) and whether the identified microbial indicators associate with the same environmental factors consistently across broad spatial and temporal scales. Further, associations between pelagic microbes and the environment are often documented as stochastic, which is partly explained by “functional redundancy” within the microbiome [23–25], whereby genes for many metabolic functions are present across broad classes of microorganisms [26–30] and microbial communities therefore likely have many compositional alternatives for carrying out the same process in any given environment. This phenomenon raises the possibility that microbial metabolic function could more reliably reflect environmental metrics than taxonomic identity, and this has been reported across plant [31, 32], soil [33], human gut [34, 35], and marine microbiomes in pelagic waters [23–25, 30, 36]. Genes for metabolic cellular functions like photosynthesis, nitrification, ammonia oxidation, sulfate reduction, and virulence have also been proposed as having higher utility in predicting environmentally induced changes that translate to shifts in reef health [10, 15, 19]. However, it is important to note that recent findings indicate that in Florida reef waters, the taxonomic microbiome (16S rRNA gene) was a stronger predictor of both physico-chemical and benthic reef properties compared to the functional microbiome (metagenome) and metabolome of the reef water [37]. This highlights the need for further research to fully understand the potential contributions of functional genes in different reef ecosystems.

Previous studies documenting community composition of reef bacterioplankton (seawater bacteria and archaea) across the Great Barrier Reef (GBR) have indicated a large influence of geography and season [19] with different explanatory drivers identified across the GBR. Using 16S rRNA gene amplicon sequencing, reef bacterioplankton of inshore GBR reefs of the Wet Tropics region were shown to predominantly respond to riverine inputs characterised by declining salinity and elevated organic and inorganic nutrients [38]. In comparison, the main drivers on inshore reefs in the central GBR were temperature, total suspended solids, particulate organic carbon, and macroalgae [11, 39]. Due to these differences in geographical sites and/or different times of sampling, potentially in addition to methodological variations in field sample collection and laboratory processing, these independent meta-omics studies have also identified somewhat inconsistent seawater microbial indicators for the same environmental metric. For example, *Rhodobacteraceae* and *Flavobacteriaceae* were identified as indicative of elevated nutrients in degraded inshore reefs of the central GBR [11, 39]; however, they were not identified as indicators of nutrient enrichment and poor water quality in the Tully River region of the northern GBR [38]. While there have been attempts to consolidate microbial community composition and environmental data sets spanning the GBR (i.e., meta-analysis by [19]), associations between reef bacterioplankton composition and nutrients were largely partitioned by cross-shelf spatial variation, with heterotrophic microbes and reduced bacterial diversity documented in inshore reefs, in contrast to more diverse and autotrophic bacterioplankton communities in oligotrophic mid- and outer-shelf GBR surface waters [19]. These findings suggest that putative indicator taxa were unique to their respective region, and may not serve as a general indicator of a specific continuous environmental metric stably across the GBR. Importantly, it remains unknown how microbial functional potential changes across the broad spatiotemporal scales of the GBR as previous studies predominantly focused on taxonomically profiling reef bacterioplankton communities through 16S rRNA gene sequencing (notable exception: [39]), which may mask variation hidden by functional redundancy. Therefore, here we measure microbial functional genes directly to assess their reliability as indicators of metrics relevant to reef health.

In this study, we perform a gene-centric analysis on surface seawater metagenomes collected from 48 offshore reefs (at ~ 5-m depth) across the length of the GBR, integrating microbial metagenomic and physico-chemical data to (1) identify stable microbial indicators—both taxonomic and functional genes—which consistently respond to specific physico-chemical variables (e.g., nutrient loads, temperature, salinity) across broad spatiotemporal scales in the GBR, and (2) to assess whether microbial taxa or functional genes exhibit greater stability in their associations with these environmental factors. To achieve objective (1), we extended a Sparse Partial Least Squares analysis (sPLS, see [40, 41]), widely used in microbial oceanography to correlate microbial data with continuous environmental metrics (see, e.g., [42–44]), with a Multivariate INTegrative method (MINT, see [45]) to integrate data from four independent sampling trips. This omics integration approach aimed to uncover microbial indicators that are stable/shared across trips, hence persistently correlating to the same physico-chemical variables across space and time in offshore GBR reefs. To achieve objective (2), we applied data perturbation with cross-validation (CV) to first quantify indicator statistical stability, measured as the reoccurrence of microbial indicator taxa or GO terms across independent CV runs, and subsequently evaluate the diagnostic potential (i.e., higher stability scores = higher diagnostic value) of microbial functional information in surpassing taxonomy for reef health assessments, which we hypothesized based on the principles of functional redundancy. Our results demonstrate the potential of reef seawater microbes to accurately inform nutrient concentrations, contributing to the potential to link seawater microbes and reef health.

## Methods

### Seawater collection and field processing

**Fig. 1:**
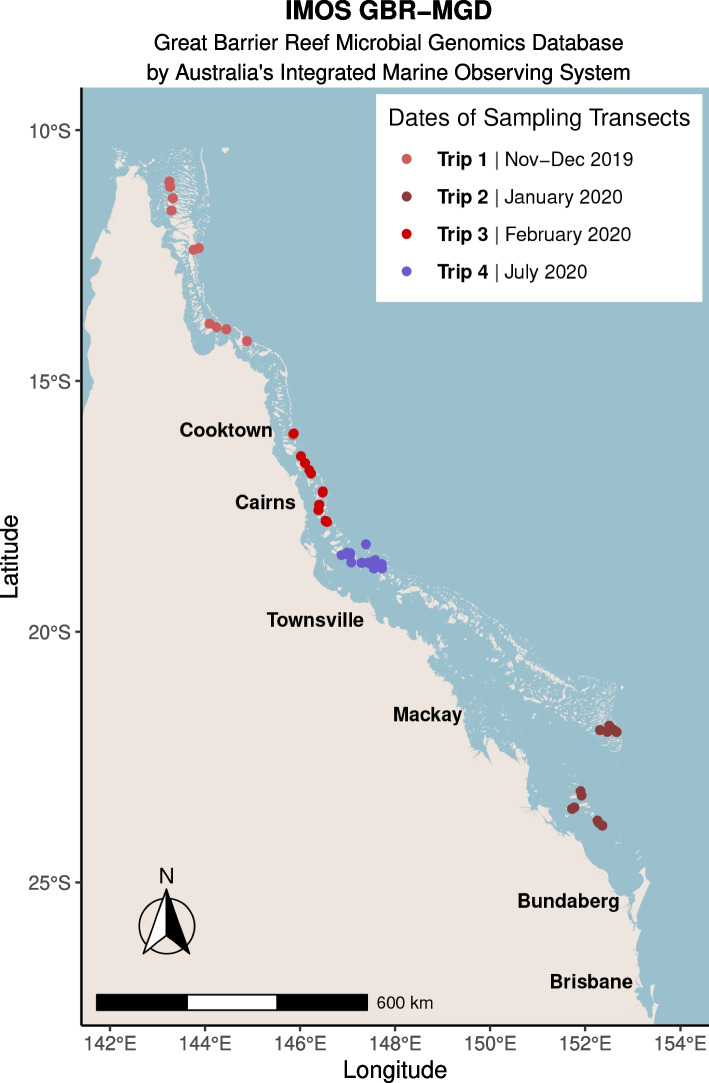
Field sampling design for the GBR-MGD (Great Barrier Reef Microbial Genomics Database) dataset by Australia's Integrated Marine Observing System (IMOS). Seawater was collected from 48 offshore GBR reef sites for microbial community metagenomic sequencing and analysis of 17 physico-chemical variables over 4 trips between November 2019 and July 2020. Reef sites are colored in red or blue tones to denote trips that occurred during the austral summer (wet season) or austral winter (dry season), respectively

Surface seawater (at 5-m depth, approximately 5–15 m from the reef benthos) was collected for water chemistry analysis and microbial community profiling at 48 reefs spanning the GBR, with each sample being collected once in time (Fig. 1). Sampling was performed from the RV Solander and RV Cape Ferguson alongside AIMS Long-Term Monitoring Program in situ reef health surveys across four trips between November 2019 and July 2020 (Fig. 1). The first three sampling trips occurred during the austral (i.e., in the Southern Hemisphere) summer (wet season) in the far northern GBR (Trip 1: November–December 2019, Cape Grenville and Princess Charlotte Bay sectors, see Fig. S1), the southern GBR (Trip 2: January 2020, Swains and Capricorn Bunker sectors, see Fig. S2), and in the central GBR (Trip 3: February 2020, Cairns and Innisfail sectors, see Fig. S3), while the last trip was performed during the austral winter (dry season) and also in the central GBR (Trip 4: July 2020, Townsville sector, see Fig. S4) (Fig. 1). The coordinates of the 48 surveyed reefs were visualized as maps in R Studio (R version 4.3.2) [46] as per the following: https://open-aims.github.io/gisaimsr/articles/examples.html, which used the following R packages as dependencies: raster (version 3.6.26) [47], tidyverse (version 2.0.0) [48], ggspatial (version 1.1.9) [49], sf (version 1.0.15) [50, 51], dataaimsr (version 1.1.0) [52], gisaimsr (version 0.0.1) (https://github.com/open-AIMS/gisaimsr), and ggrepel (version 0.9.5) [53].

Triplicate 5-L seawater samples were collected using Niskin bottles or by divers for analysis of water chemistry variables. A total of 14 water chemistry variables were measured using established methods [54], including ammonia (NH_4_^+^), nitrite (NO_2_^+^), nitrate (NO_3_^+^), total dissolved nitrogen (TDN), phosphate (PO_4_^3−^), total dissolved phosphorus (TDP), dissolved organic carbon (DOC), silicate (Si), total suspended solids (TSS), chlorophyll *a* (Chl-*a*), phaeophytin *a* (Phaeo), particulate organic carbon (POC), particulate nitrogen (PN), and particulate phosphorus (PP). Samples for dissolved nutrient (NH_4_^+^, NO_2_^+^, NO_3_^+^, PO_4_^3−^ — hereinafter written without specifying the electron charge for clarity, as well as Si, TDN, TDP, and DOC) analysis were immediately filtered through a 0.45-µm syringe filter (Sartorius Minisart N) into 10-mL acid-washed vials, which were pre-rinsed three times with filtered site seawater. Dissolved inorganic (NH_4_, NO_2_, NO_3_, PO_4_) and total dissolved (TDN, TDP) samples were stored frozen (− 18 °C) until analysis. Samples for DOC analysis were acidified with 100 µL of AR-grade hydrochloric acid; DOC and Si samples were stored refrigerated (4 °C) until analysis. Samples for particulate nutrient (POC, PN, PP) and Chl-*a* analysis were manifold filtered through pre-combusted (450 °C for 4 h) 25-mm diameter filters (Whatman GF/F, nominal pore size 0.7 µm), folded, placed in pre-combusted aluminum foil envelopes, and stored frozen (− 18 °C) until analysis. Samples for TSS analysis were manifold filtered onto pre-weighed 47-mm diameter polycarbonate filters (GE Water & Process Technologies, pore size 0.4 µm), which were then triple rinsed with ultrapure water to remove residual salt from the filter. TSS filters were stored at room temperature while onboard the vessel and were immediately placed in a drying oven (60 °C) overnight upon return to AIMS (Townsville, Queensland).

In addition to the water chemistry variables listed above, temperature, salinity, and Chl-*a* fluorescence measurements were also retrieved from the underway sampling systems on the RV Solander and RV Cape Ferguson, which are part of Australia’s Integrated Marine Observing System (IMOS) Ships of Opportunity Sensors on Tropical Research Vessels sub-facility [55]. Temperature and salinity data were measured at 10-s intervals using a SBE 38 digital oceanographic thermometer and SBE 21 SeaCAT Thermosalinograph (Sea-Bird Scientific), while fluorescence was measured using an ECO-FLNTU-RT (WET Labs). Intake depths for underway systems were 1.9 m (RV Cape Ferguson) and 2.5 m (RV Solander). For temperature, salinity, and Chl-*a* fluorescence, a single value that was closest to the sampling time was recorded at each site. Hereinafter, we use the term “physico-chemical variables” to encompass the 17 variables measured in this study, which include 14 water chemistry variables, as well as temperature, salinity, and Chl-*a* fluorescence.

Seawater for metagenomic sequencing was collected concurrently with water chemistry samples in four 5-L replicates. Collected seawater was immediately passed through a 5-µm Minisart® NML syringe prefilter (Sartorius, Goettingen, Germany) to remove large debris and eukaryotic cells and subsequently through a 0.22-µm Millipore® Sterivex-GP™ Pressure Filter (Merck Millipore, Darmstadt, Germany) using a peristaltic pump on board the research vessel. The Sterivex filters were snap-frozen in liquid nitrogen and stored at − 75 °C until processed in the laboratory.

### Laboratory processing for water chemistry and metagenomic sequencing

Laboratory analyses of water chemistry samples were conducted at the AIMS Analytical Technology and Water Quality Laboratories within one month (Chl-*a*, DOC, and TSS) or three months (all other variables) of collection. Inorganic dissolved nutrient concentrations (NH_4_, NO_2_, NO_3_, PO_4_, Si) were determined using standard wet chemical methods [56–58] on a Seal AA3 segmented flow analyzer. Total dissolved samples (TDN, TDP) were persulfate digested [59] and analyzed for inorganic concentrations as above. Concentrations of DOC, POC, and PN were determined via high temperature catalytic combustion using a Shimadzu TOC-L carbon analyzer with a solid sample module (SSM-5000A) for POC filters and a nitrogen module (TNM-L) for PN filters. Concentration of PP was determined spectrophotometrically [57] following digestion in hot acid persulfate [60]. Concentration of Chl-*a* was determined by grinding filters in 90% acetone (with a 2-h incubation period in the dark) and reading the supernatant on a fluorometer (Turner Designs 10AU); samples were then acidified and reread to determine the concentration of Phaeo and correct Chl-*a* measurements for its interference [61]. Concentrations of TSS were determined gravimetrically based on pre- and post-sampling filter weights.

DNA was extracted from 0.22-µm Sterivex filters using a phenol:chloroform:isoamyl alcohol extraction with ethanol precipitation (as in [62], with the addition of 18 µL (100 mg mL^−1^) lysozyme to the lysis buffer. DNA was quality-checked with a NanoDrop 2000 spectrophotometer (Thermo Fisher Scientific, Australia) and quantified using a Qubit 3 fluorometer (Thermo Fisher Scientific, Australia) before submission for Illumina Nextera Flex sequencing using the NovaSeq at Microba Life Sciences Ltd. (Brisbane, QLD, Australia). An average of 17,464,769 ± 4,075,366 of 150-bp reads was sequenced from each of the 191 samples (47 sites × four replicates at each site and three replicates at Hedley Reef) (Table S1). The three negative controls had a low number of sequenced reads (173,749 ± 49,755; Table S1).

### Metagenomic data processing

A read-based metagenomics analysis was applied to separate taxonomic and functional profiling of seawater microbiomes in offshore GBR reefs and elucidate the role of environmental filtering and functional redundancy in shaping reef bacterioplankton communities separately at taxonomic and functional levels (see [23, 25]). Demultiplexed raw reads were first quality-checked in FastQC (version 0.11.3) [63] and quality-filtered in Trimmomatic (version 0.38) [64] to trim barcodes/adapters and remove low-quality bases (Phred < 20). In total, 78.84% of reads were retained after quality filtering in Trimmomatic (an average of 13,853,993 ± 3,324,976 reads per sample) (Table S1). Quality-filtered reads were then aligned against the NCBI nr database using the DIAMOND (version 2.0.9) alignment tool [65]. For each read, the top match reported by Diamond with *e*-value of < 10^−5^ was retained to exclude poor annotations. Resulting Diamond files (in daa format) were then imported into MEGAN (version 6.23.0) [66] for community profiling. Raw microbial abundance counts were exported from MEGAN for genus-level taxonomic and functional (GO terms) composition and subsequently imported into R Studio (version 4.3.2) [46] using the phyloseq (version 1.46.0) R package [67]. Using R, further filtering steps included the removal of (1) non-annotated reads, taxa annotated as (2) eukaryotic (774 hits) or (3) viral (35 hits), and removal of (4) prokaryotic reads annotated to the domain level only (Bacteria or Archaea), leaving 48% of the total data set. The last filtering step included the removal of (5) rare/spurious reads (relative abundance < 0.0001%), resulting in a total of 621 of the initial 1257 prokaryotic taxa (collapsed at genus level or above) for the final dataset on microbial taxonomy, while for gene annotation dataset, this filtering resulted in 4287 GO terms. This gave a final range of sequences of 3,752,207 ± 1,402,666 per sample (Table S2). Microbial abundance data was then center log ratio (CLR) normalized in the microbiome (version 1.24.0) R package [68] to account for sparsity and compositional nature of microbial metagenomic sequencing data. Pseudo counts were introduced prior to CLR normalization as log 0 is undefined. These CLR-transformed counts or relative abundance data were used in downstream statistical analysis and visualisation in R Studio. Final composite plots were made in Inkscape 0.92.5.

### Summarizing water chemistry data and microbial community data

Principal Components Analysis (PCA) was applied in the R package mixOmics (version 6.26.0) [69] as an unsupervised approach to visualize the main clustering patterns between reef sites based on physico-chemical variables. The number of optimal PCA components was determined using the mixOmics *tune.pca()* function. The PCA biplot was complemented with a heatmap to visualize the level of change in physico-chemical variables in more detail, across each reef site, by centering (median = 0) and scaling (standard deviation (SD) = 1) each of the 17 physico-chemical variables across sites.

PCA was used to visualize the main clustering patterns of reef sites based on seawater microbial communities (both for microbial taxonomy and GO terms, using CLR-normalized counts to account for compositionality and sparsity of metagenomics sequencing data), following the same approach as detailed in the previous paragraph. Pairwise permutational multivariate analysis of variance (PERMANOVA), implemented in the *pairwise.adonis()* R wrapper function [70], was applied to test if distances between PCA (computed for microbial taxonomy) group centroids (i.e., between the four trips) were statistically significant. Stacked bar charts were used to visualize microbial taxonomy profiles collapsed at (1) genus level (by showing the top 20 most abundant microbial genera), (2) at phylum level, and (3) at genus level but only within phylum *Bacteroidetes* which increased in relative abundances during summer. Microbial diversity was also compared between the four trips by computing a Shannon index (1) for the overall community profiles and (2) only within phylum *Bacteroidetes*. Shannon diversity results were visualized as boxplots, and the variation in alpha-diversity scores across trips was compared with pairwise Wilcoxon Rank-sum tests in R, which were integrated within microbial diversity boxplots.

### Integrating microbial and physico-chemical data

Partial (geographic distance-corrected) Mantel tests with 10,000 permutations and Bonferroni correction were applied to identify physico-chemical variables that significantly correlated with seawater microbial communities (see [25, 71]). In the partial Mantel tests, Bray–Curtis dissimilarities were computed within partial Mantel tests from relative abundances of microbial data with Euclidean distances of physico-chemical variables, while controlling for the effect of geography by including a third distance matrix of spatial distances between reef sites, expressed in km. A total of 34 partial Mantel tests were computed for both the taxonomy and functional genes datasets with each of the 17 physico-chemical variables.

Indicator microbes and GO terms were identified for each of the 17 physico-chemical variables using MINT sPLS — Multivariate INTegration Sparse Partial Least Squares [40, 41, 45, 69]. sPLS [40, 41] fits a linear relationship between multiple predictors (physico-chemical variables) with multiple continuous responses (microbial taxa or GO terms), while MINT [45] is based on multigroup PLS that includes information about samples belonging to independent subsets of samples (i.e., sampling trips). In this context, MINT sPLS integrated samples from independent subsets to remove unwanted sources of variation due to trips (i.e., confounding effects between season and geography), identifying microbial indicator taxa and GO terms that are shared/universal across the sampling trips. Prior to correlating metagenomic and physico-chemical data in MINT sPLS, median values per reef site were computed for each of the 17 physico-chemical variables as the number of Niskin deployments differed for molecular (4 replicates) and water chemistry (3 replicates) sampling. MINT sPLS selected 100 key features (i.e., seawater microbial taxa and GO terms, spanned across the first two MINT sPLS dimensions, with 50 features per dimension) that show the highest covariance with the 17 physico-chemical variables. MINT sPLS partial correlations were visualized as heatmaps for indicator taxa and GO terms using mixOmics [69].

A Leave-one-group-out cross-validation (LOGOCV) [45] was applied to investigate the stability of microbial indicator taxa/GO terms identified in MINT sPLS dimension 1 across sampling trips. LOGOCV performed cross-validation (CV) where one CV fold equals one study (sampling trip), hence four times until each of the four sampling trips was left out once. Indicator taxa/GO terms shared across different sampling trips were assigned stability scores of either 1 (selected in each of the four LOGOCV iterations), 0.75 (selected in 3/4 of the LOGOCV iterations), or 0.5 (selected in 2/4 of the LOGOCV iterations). A stability score of 0.25 indicates trip-specific microbiome/environment associations being identified in 1/4 LOGOCV iterations; hence, these indicators were considered unstable (i.e., not shared across sampling trips). These stability scores were integrated with MINT sPLS heatmaps as barplots, visualized in the ggplot2 (version 3.5.1) R package [72].

### Comparing the potential of microbial *indicator* taxa and genes to infer reef physico-chemical metrics

The Bray–Curtis similarity index (expressed as 1 — Bray–Curtis dissimilarity, computed with the *vegdist()* function in vegan (version 2.6.4) R package, see [73]) was used to compare within-site similarity (0 = dissimilar, 1 = identical) of reef bacterioplankton communities at functional and taxonomic levels. Bray–Curtis similarity scores were computed within each of the 48 reefs and at various hierarchical levels, both for microbial taxonomy (genus, family, order, class, and phylum) and functions (GO terms collapsed at levels 5, 4, and 3). For each of these levels, Bray–Curtis similarity scores (0 — low similarity; 1 — high similarity) were visualized as boxplots, with the higher similarity scores being indicative of the lower community variability in the microbiome composition within one reef site.

To identify if microbial indicator taxa or GO terms associate more robustly with physico-chemical variables in the surrounding reef, we used the same principles presented for MINT sPLS (i.e., inferring indicator stability using LOGOCV), but instead of removing one group during LOGOCV iterations (samples belonging to one trip), within each CV iteration, a random subset of samples from each trip was removed as a single subset of data. In more detail, sPLS was applied within each of the trips to account for confounding effects of geography and time, with microbial taxa and GO terms selected as predictor datasets and physico-chemical variables as the response dataset. This resulted in a total of eight sPLS models (four trips × two datasets, for microbial taxa and GO terms). For each of the eight sPLS models, a fourfold CV with 50 repeats was applied to assess reproducibility of the microbiome/environment signatures when the training set was subsampled via cross-validation, and each of the 50 indicator taxa/GO terms selected by sPLS on component 1 were assigned a stability score averaged across the 200 CV runs (fourfold CV × 50 iterations), ranging from 0 (i.e., low stability) to 1 (i.e., high stability). These stability scores were visualized as boxplots, and the variation between stability scores from indicator taxa and GO terms (within each of the four sampling trips) was tested with a Wilcoxon rank-sum test in R, which were integrated within stability boxplots.

## Results

### Higher nutrients in GBR surface waters during summer

**Fig. 2:**
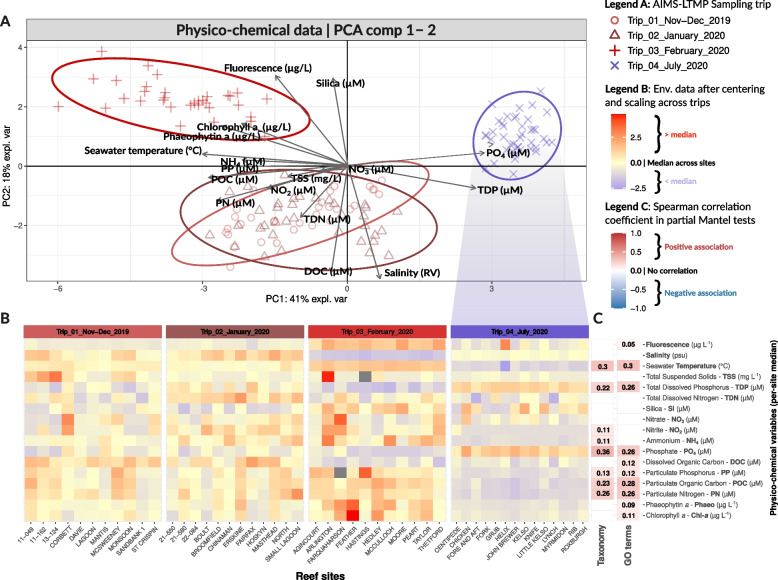
Summarizing water chemistry data and identifying drivers of seawater microbial communities. **A** Principal Components Analysis (PCA) shows the main clustering patterns of reef sites based on physico-chemical variables. Reef sites use specific shapes and are colored in red or blue tones to denote trips that occurred during the austral summer (wet season) or austral winter (dry season), respectively. **B** The heatmap shows changes in physico-chemical variables (*y*-axis) across the reef sites (*x*-axis). Physico-chemical variables were centered (median = 0) and scaled (standard deviation (SD) = 1) across reef sites, and values that deviate from the median (0) were shown in red (> median) and blue (< median). Two instances of missing water chemistry measurements were indicated by grey rectangles. **C** A total of 34 partial Mantel tests (corrected for geographic distance) were conducted for each of the 17 physico-chemical variables and for both microbial datasets on taxonomy and GO terms. Nonsignificant results (*p*-value > 0.05, Bonferroni correction) are shown as white cells, while colored cells denote statistically significant trends (*p*-value < 0.05, Bonferroni correction), indicating positive (red) or negative (blue) associations (Spearman’s rank correlation coefficients *ρ* shown as the numeric value) between microbial and environmental distance matrices, while corrected for geographic distance between reefs (expressed in km)

To identify drivers of microbial community variation for reef bacterioplankton (Fig. 2, Table 1), a total of 17 physico-chemical variables were derived from seawater samples from 48 offshore reefs across the length of the GBR (Fig. 1), including temperature, salinity, fluorescence, and particulate and dissolved nutrients. The largest source of variation in reef water chemistry was the timing of sampling trips across the austral summer or winter periods (41% of explained variance, PCA dimension 1), with samples collected in the peak of summer (Trip 3 — February 2020; SST = 30.16 ± 0.39 °C) additionally separating from early summer sampling trips (Trip 1 — Nov-Dec 2019, SST = 27.78 ± 0.43 °C and Trip 2 — January 2020, SST = 27.16 ± 0.61 °C; 18% of explained variance, PCA dimension 2) (Fig. 2a, Table 1, Fig. S5). Overlaying physico-chemical data in a PCA visualization showed that summer trips 1–3 were characterized by elevated temperature (median of 28.30 ± 1.51 °C across summer Trips 1–3 vs 24.4 ± 0.95 °C in winter trip 4), and higher concentrations of particulate nutrients which were on average threefold higher in comparison to the winter trip (*PP* = 0.06 ± 0.01 µM for summer Trips 1–3 vs 0.02 ± 0.01 µM in winter Trip 4, ~ 3.4-fold increase in summer; PN = 1.27 ± 0.05 µM vs 0.50 ± 0.10 µM, ~ 2.5-fold increase in summer; POC = 8.54 ± 1.25 µM vs 3.67 ± 1.00 µM, ~ 2.3-fold increase in summer) (Fig. 2a, b, Table 1, Fig. S5). Chlorophyll fluorescence, Chl-*a*, and Phaeo were highest at sites collected in the central GBR in February 2020 (fluorescence = 0.32 ± 0.05 µg L^−1^, Chl-a = 0.23 ± 0.18 µg L^−1^, and Phaeo = 0.36 ± 0.15 µg L^−1^; Fig. 2a, b, Table 1, Fig. S5). In contrast, reefs sampled in the austral winter had a twofold increase in dissolved phosphorus (PO_4_ = 0.09 ± 0.02 µM and TDP = 0.26 ± 0.02 µM) in comparison to the summer trips 1–3 (PO_4_ = 0.04 ± 0.01 µM and TDP = 0.20 ± 0.03 µM) (Fig. 2a, b, Table 1, Fig. S5). Notably, chemistry profiles of samples collected in the early austral summer were comparable despite being > 1500 km apart in the far north (Cape Grenville and Princess Charlotte Bay sectors) and far south (Swains and Capricorn Bunker sectors) of the GBR, whereas samples collected during the peaks of austral summer and winter were the most distinct, although they were geographically close in the central GBR (~ 200 km apart, Cairns and Innisfail sectors for austral summer samples and Townsville sector for austral winter samples). This highlights that water chemistry measurements in offshore GBR surface waters are predominantly driven by seasonality and less influenced by geography.

**Table 1:** Physico-chemical data. Median ± SD values of 17 physico-chemical variables (rows) collected across 48 offshore GBR reefs. The values are collapsed across four sampling trips (columns)

**Physico-chemical variables**	Trip 1 (median ± SD)	Trip 2 (median ± SD)	Trip 3 (median ± SD)	Trip 4 (median ± SD)
Chlorophyll a (µg L^−1^) — **Chl-*a***	0.18 ± 0.06	0.16 ± 0.08	0.32 ± 0.18	0.11 ± 0.03
Phaeophytin (µg L^−1^) — **Phaeo**	0.18 ± 0.04	0.20 ± 0.08	0.36 ± 0.15	0.10 ± 0.02
Particulate nitrogen (µM) — **PN**	1.23 ± 0.35	1.27 ± 0.46	1.32 ± 0.22	0.50 ± 0.10
Particulate organic carbon (µM) — **POC**	8.06 ± 2.86	7.60 ± 1.89	9.95 ± 2.29	3.66 ± 1.00
Particulate phosphorus (µM) — **PP**	0.05 ± 0.02	0.05 ± 0.02	0.07 ± 0.03	0.02 ± 0.01
Dissolved organic carbon (µM) — **DOC**	84.51 ± 5.99	81.92 ± 9.89	67.22 ± 4.60	69.30 ± 4.67
Phosphate (µM) — **PO₄**	0.05 ± 0.03	0.04 ± 0.02	0.02 ± 0.02	0.09 ± 0.02
Ammonium (µM) — **NH₄**	0.39 ± 0.16	0.58 ± 0.27	0.74 ± 0.44	0.12 ± 0.06
Nitrite (µM) — **NO₂**	0.03 ± 0.02	0.04 ± 0.01	0.04 ± 0.02	0.01 ± 0.01
Nitrate (µM) — **NO₃**	0.30 ± 0.25	0.33 ± 0.15	0.35 ± 0.31	0.23 ± 0.16
Silica (µM) — **Si**	1.41 ± 0.30	1.30 ± 0.44	2.10 ± 0.55	1.78 ± 0.65
Total dissolved nitrogen (µM) — **TDN**	5.47 ± 0.83	6.62 ± 0.82	5.64 ± 0.72	5.18 ± 0.75
Total dissolved phosphorus (µM) — **TDP**	0.20 ± 0.03	0.23 ± 0.04	0.16 ± 0.03	0.26 ± 0.02
Total suspended solids (mg L^−1^) — **TSS**	0.48 ± 0.41	0.15 ± 0.15	0.36 ± 0.52	0.11 ± 0.10
Temperature (°C)	27.78 ± 0.43	27.13 ± 0.61	30.01 ± 0.39	24.22 ± 0.95
Salinity (psu)	35.35 ± 0.21	35.52 ± 0.17	34.71 ± 0.05	35.16 ± 0.04
Chl-*a* fluorescence (µg L^−1^)	0.10 ± 0.01	0.10 ± 0.02	0.34 ± 0.05	0.13 ± 0.12

### Microbial community composition and functional gene profiles differ by season

**Fig. 3:**
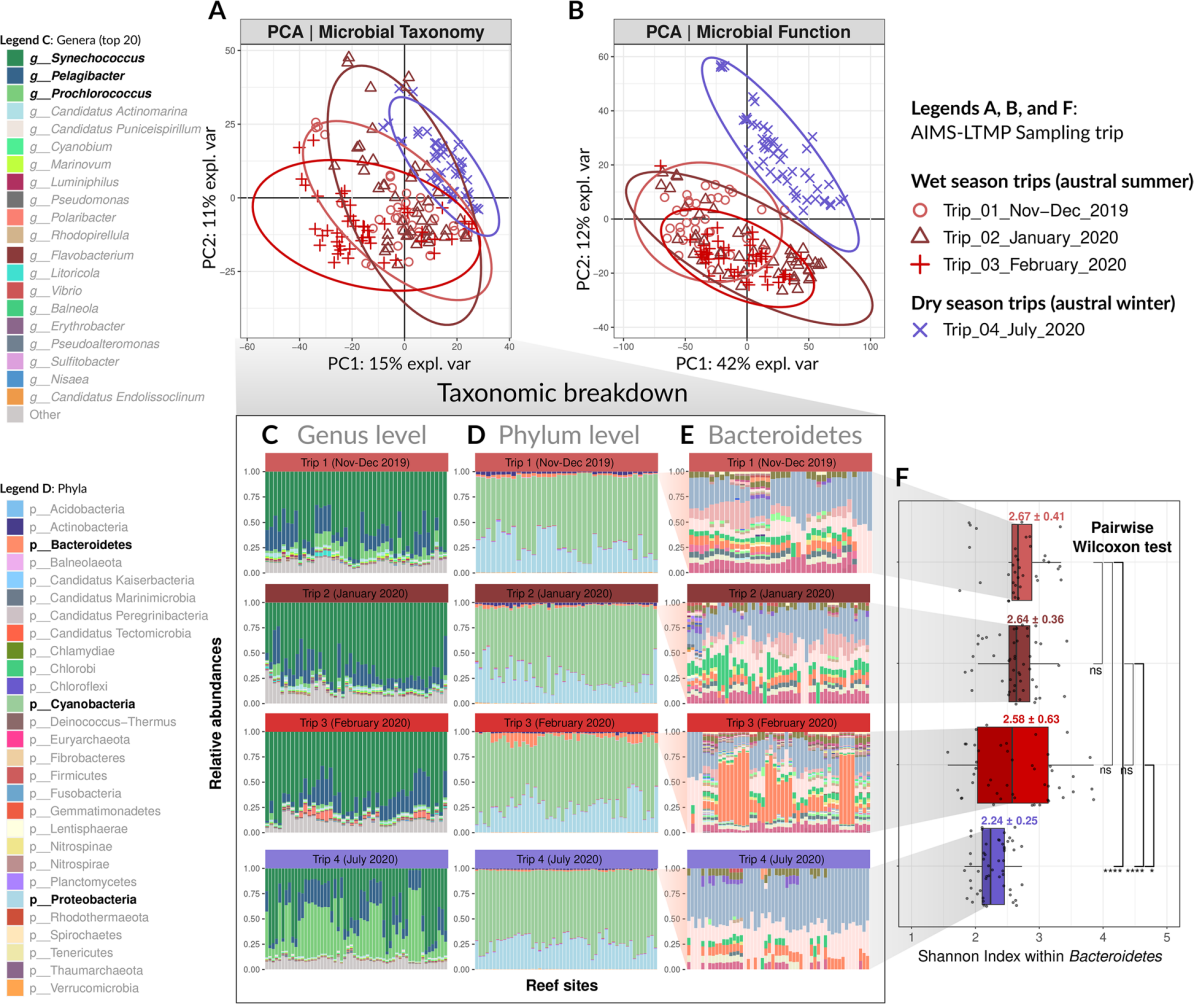
Main clustering patterns of seawater microbial communities. Principal Components Analysis (PCA) plots show the main clustering patterns of reef sites based on microbial community composition, both for microbial taxonomy (**A**) and microbial GO terms (**B**). Reef sites are colored in red or blue tones to denote trips that occurred during the austral summer (wet season) or austral winter (dry season), respectively. Stacked barplots illustrate microbial relative abundances (*y*-axis) for each sample (*x*-axis), with reef sites grouped by their corresponding sampling trip. These barplots represent the following: **C** the top 20 most abundant microbial genera, **D** all 29 identified microbial phyla, and **E** all microbial genera within the phylum *Bacteroidetes*. The top three most abundant genera (**C**) and phyla (**D**) are highlighted in bold, and the legend for genera within *Bacteroidetes* (**E**) was excluded due to the large number of taxa. **F** Boxplots illustrate microbial diversity (Shannon index) for genera within phylum *Bacteroidetes*, across sampling trips. The symbols *, **, ***, and **** denote levels of statistical significance in pairwise Wilcoxon rank-sum tests when testing variation of *Bacteroidetes* Shannon diversity scores across the four sampling trips: * for *p* < 0.05, ** for *p* < 0.01, *** for *p* < 0.001, and **** for *p* < 0.0001, indicating increasing levels of significance. “ns” indicates nonsignificant results, where *p* ≥ 0.05

A total of 29 bacterial and archaeal phyla were identified in the seawater communities of the 48 surveyed offshore GBR reefs, of which three dominant phyla, *Cyanobacteria* (average 68% relative abundance), *Proteobacteria* (26%), and *Bacteroidetes* (2.6%), together comprised an average of 96.6% relative abundance of retrieved sequences (Fig. 3d). At the genus level, three genera dominated the seawater communities: *Synechococcus* with 54.99% average relative abundance, *Pelagibacter* (also known as SAR11) at 15.89% relative abundance, and *Prochlorococcus* at 11.93% (Fig. 3c).

PCA showed that seawater microbial communities differed between seasons, with samples primarily clustered by collections during the austral summer (Trips 1–3) vs winter (Trip 4) with around 26% of total variance attributable to the first two principal components (Fig. 3a). PCA clustering was supported by pairwise PERMANOVA indicating that seawater community composition significantly differed when comparing summer vs winter trips (*p* < 0.05, Bonferroni correction) but not between summer trips (Table 2). These differences in community composition were primarily driven by increased relative abundances of *Prochlorococcus* during the winter trip (average 32.93% vs 3.23% relative abundance in summer trips) and decreased *Synechococcus* (average 37.02% in winter vs 62.38% in summer trips) (Fig. 3c). Several members within the *Bacteroidetes* phylum were also more dominant in the three summer trips (mean relative abundances for summer trips: Trip 1 = 2.13%, Trip 2 = 1.80%, Trip 3 = 5.31% and the winter Trip 4 = 1.14%, see Fig. 3d, e), particularly the family *Flavobacteriaceae* which were the most discriminatory taxa in samples collected during the peak of summer in February 2020 (Fig. 3e, Fig. S7). Apart from increasing in relative abundance, members of the *Bacteroidetes* phylum were also more diverse during the summer trips (median Shannon index for Trip 1 = 2.67 ± 0.41, Trip 2 = 2.64 ± 0.36, and Trip 3 = 2.58 ± 0.63) compared to the winter (Trip 4: median Shannon index 2.24 ± 0.25) (Fig. 3e, f), with pairwise Wilcoxon rank-sum tests only being significant (*p* adjusted < 0.05) for summer/winter trip comparisons (Fig. 3f). When comparing the Shannon index computed for overall microbial communities, we identified no significant difference (*p* adjusted > 0.05, Wilcoxon rank sum test) in median Shannon diversity between trips (Fig. S6, Table S3, Table S4).

**Table 2:** The pairwise permutational multivariate analysis of variance (PERMANOVA) test for microbial communities (taxonomic level). Significant results (*p*-value < 0.05, Bonferroni correction) are highlighted in bold

Pairwise comparison		SumSqrs	MeanSqrs	F	*R*^2^	*p*-value	*p*-value (Bonferroni corrected)
Trip 1	Trip 2	0.111	0.111	3.168	0.037	0.057	0.344
Trip 1	Trip 3	0.203	0.203	5.765	0.066	0.009	0.055
Trip 1	**Trip 4**	1.856	1.856	28.370	0.248	0.000	**0.001**
Trip 2	Trip 3	0.091	0.091	2.688	0.028	0.076	0.453
Trip 2	**Trip 4**	2.933	2.933	48.630	0.332	0.000	**0.001**
Trip 3	**Trip 4**	3.210	3.210	52.889	0.353	0.000	**0.001**

Microbial functional profiles (GO terms) were also primarily clustered by sampling during the austral summer (Trips 1–3) vs winter (Trip 4), although with stronger separation compared with taxonomic composition (54% of variance attributable to the first two PCA dimensions vs. 26%) (Fig. 3b). Seawater microbial communities collected during summer Trips 1–3 were characterized by elevated transporters (i.e., ABC transporters, TRAP transporter permease proteins, and UAA transporters, as well as various ion transporters) and GO terms encoding for oxidative phosphorylation (NADH:ubiquinone oxidoreductase, complex 1 of the respiratory chain), which were comparatively underrepresented in samples collected in the winter Trip 4 (Fig. S8).

### Particulate and dissolved nutrients drive seawater microbial community composition

Partial Mantel tests identified nine and 11 physico-chemical variables which were associated with taxonomic composition and gene-based microbial profiles respectively, while accounting for geographic distance between reefs (*p* < 0.05, Bonferroni correction; Fig. 2c). The highest Spearman’s rank correlation coefficients (*ρ*, ranging from − 1 to 1, with negative values indicating an inverse relationship and positive values denoting the same trajectory), and therefore the strongest physico-chemical variables influencing both taxonomic composition and functional profiles, were computed for phosphate (*ρ* = 0.36 for microbial taxonomy and *ρ* = 0.26 for microbial functional genes), seawater temperature (*ρ* = 0.3 and 0.3), and particulate nutrients (POC: *ρ* = 0.23 and 0.28; PN: *ρ* = 0.26 and 0.26; PP: *ρ* = 0.13 and 0.12) (Fig. 2c). Physico-chemical variables that were significantly associated only to microbial genes but not microbial taxonomy included Chl-*a* (*ρ* = 0.11), Phaeo (*ρ* = 0.09), fluorescence (*ρ* = 0.05), and DOC (*ρ* = 0.12). In contrast, NH_4_ (*ρ* = 0.11) and NO_2_ (*ρ* = 0.11) positively associated only to microbial taxonomy, but not functional gene profiles (Fig. 2c). Only positive Spearman correlations were calculated for the physico-chemical variables significantly associated with reef bacterioplankton, indicating that both taxonomic and functional composition of seawater microbes become increasingly dissimilar as associated physico-chemical variables change. This suggests that seawater microbes exhibit a deterministic response to their surrounding environment, with microbial population dynamics or community structure being directly influenced by specific nutrient conditions and changing in proportion to variations in measured nutrients.

**Fig. 4:**
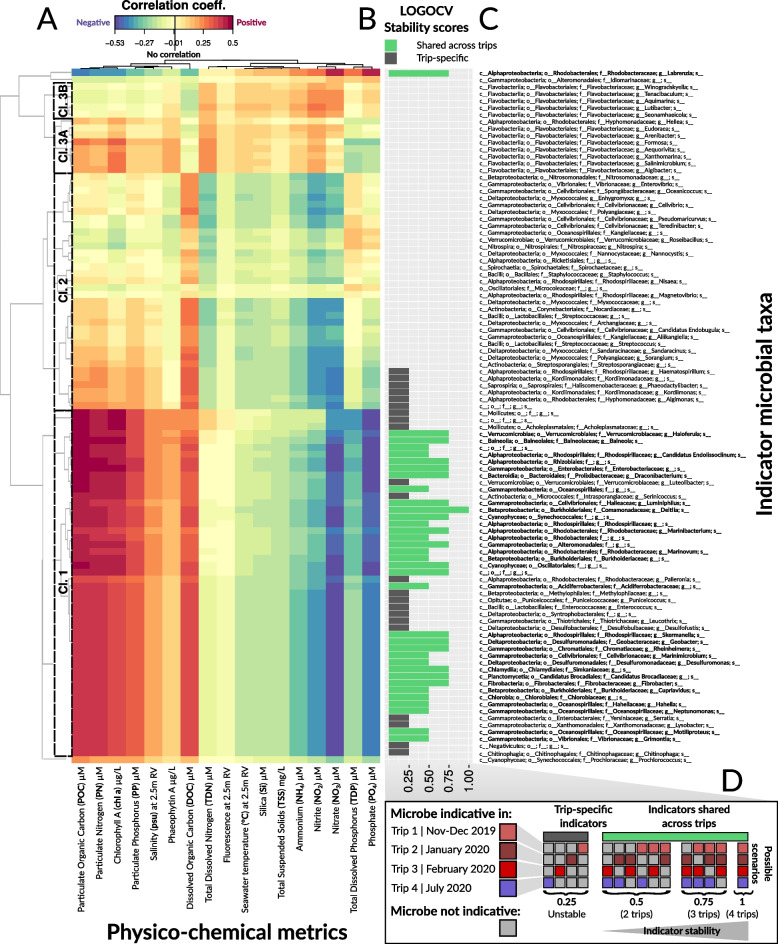
MINT sPLS — associations between microbial taxa and physico-chemical variables. **A** The heatmap shows similarity values (partial correlations) between 17 continuous physico-chemical variables (predictor dataset) and 100 microbial taxa (response dataset) selected across the first two MINT sPLS dimensions. Heatmap cells are colored to indicate either positive (red) or negative (blue) correlation. Heatmap rows and columns were clustered with a complete Euclidean distance method, with three clusters highlighted with a dashed line and numbered as they were discussed in the text. **B** Indicator stability barplots as determined by leave-one-group-out cross-validation — LOGOCV. Microbial indicator taxa are colored in green if they are shared across sampling trips or in gray if they are trip-specific. **C** Taxonomic breakdown of indicator microbes, with indicator taxa shared across different sampling trips (as inferred by LOGOCV) further highlighted in bold. **D** Explanation of LOGOCV stability scores through 15 possible scenarios. Indicator microbes are assigned colors if indicative in a particular trip (with colouring scheme for trips corresponding to Fig. 1), while non-indicator taxa are colored in gray (**D**, left). The lowest LOGOCV stability score of 0.25 indicates trip-specific microbial indicators (selected in 1/4 LOGOCV iterations, with four possible scenarios), which were therefore considered unstable as these indicators are not reproducible across sampling trips (**D**, middle). Stable microbial indicators (shared across trips) were assigned LOGOCV stability scores of either 0.5 (selected in 2/4 of the LOGOCV iterations, with six possible scenarios), 0.75 (selected in 3/4 of the LOGOCV iterations, with four possible scenarios), or 1, which indicated the highest indicator stability score (selected in each of the four LOGOCV iterations) (**D**, right). Only shared microbial indicator taxa (with LOGOCV stability scores of 0.5, 0.75, and 1) were considered in downstream interpretation and discussion of results

**Fig. 5:**
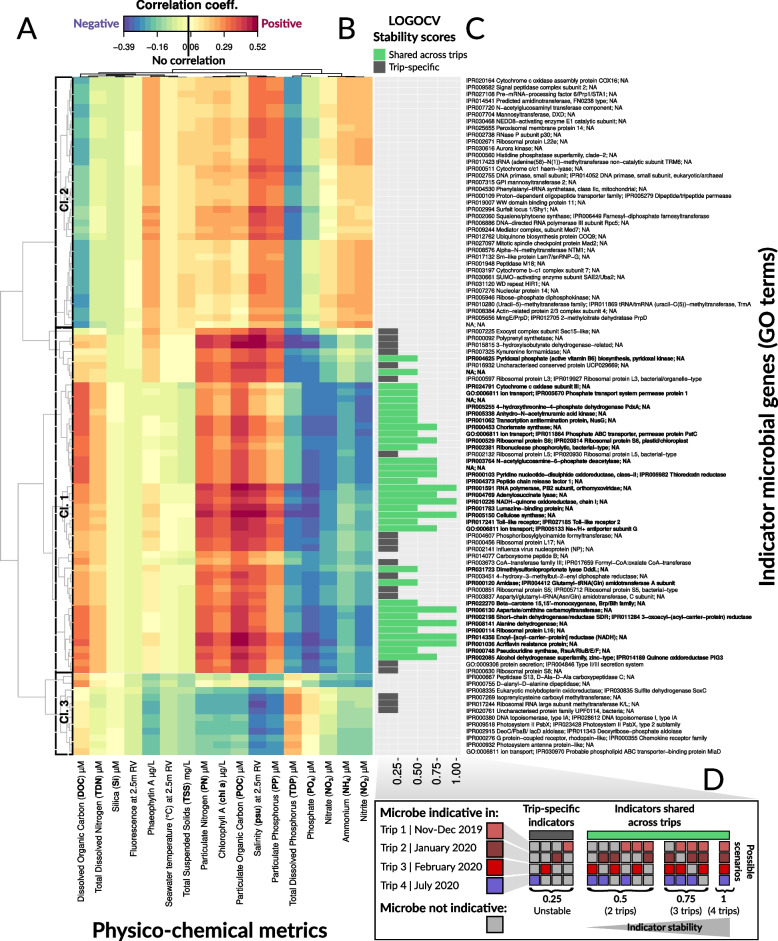
MINT sPLS — associations between microbial genes/functions (GO terms) and physico-chemical variables. **A** The heatmap shows similarity values (partial correlations) between 17 continuous physico-chemical variables (predictor dataset) and 100 microbial GO terms (response dataset) selected across the first two MINT sPLS dimensions. Heatmap cells are colored to indicate either positive (red) or negative (blue) correlation. Heatmap rows and columns were clustered with a complete Euclidean distance method, with three clusters highlighted with a dashed line and numbered as they were discussed in the text. **B** Indicator stability barplots as determined by leave-one-group-out cross-validation — LOGOCV. Microbial indicator genes are colored in green if they are shared across sampling trips or in gray if they are trip-specific. **C** GO functional annotation of indicator genes/functions, with indicator GO terms shared across different sampling trips (as inferred by LOGOCV) further highlighted in bold. **D** Explanation of LOGOCV stability scores through 15 possible scenarios. Indicator genes are assigned colors if indicative in a particular trip (with coloring scheme for trips corresponding to Fig. 1), while non-indicator genes are colored in gray (**D**, left). The lowest LOGOCV stability score of 0.25 indicates trip-specific microbial indicators (selected in 1/4 LOGOCV iterations, with four possible scenarios), which were therefore considered unstable as these indicators are not reproducible across sampling trips (**D**, middle). Stable microbial indicators (shared across trips) were assigned LOGOCV stability scores of either 0.5 (selected in 2/4 of the LOGOCV iterations, with six possible scenarios), 0.75 (selected in 3/4 of the LOGOCV iterations, with four possible scenarios), or 1, which indicated the highest indicator stability score (selected in each of the four LOGOCV iterations) (**D**, right). Only shared microbial indicator genes (with LOGOCV stability scores of 0.5, 0.75, and 1) were considered in downstream interpretation and discussion of results

Using Multivariate INTegration Sparse Partial Least Squares (MINT sPLS) to identify which indicator microbial taxa and GO terms consistently associated with the same physico-chemical variables in more than one sampling trip, we selected 100 key indicator seawater microbial taxa and GO terms (spanned across the first two MINT sPLS dimensions, with 50 features per dimension) that show the highest associations with 17 physico-chemical variables stably across trips. Since low MINT sPLS correlation scores (i.e., below the absolute value of 0.22) were observed for the 50 indicator microbial taxa and genes selected on the second MINT sPLS dimension, a leave-one-group-out cross-validation (LOGOCV) was applied to mine for stable indicators selected only on MINT sPLS dimension 1, ultimately identifying 33 microbes and 34 GO terms that are shared across two, three, or four trips (i.e., indicators assigned LOGOCV stability scores of 0.5, 0.75, and 1, respectively). All 100 indicator features (microbes in Fig. 4 and GO terms in Fig. 5) were then grouped into three “community-type” clusters based on Euclidean distance clustering (marked with dashed lines), and the clusters containing the 33 microbes and 34 GO terms as stable indicators were termed “Cluster 1” to highlight their importance and were the main focus in results interpretation and discussion. Microbial indicators in MINT sPLS clusters 2 (34 indicator taxa in Fig. 4, Cl. 2, and 37 indicator GO terms in Fig. 5, Cl. 2) and 3 (13 indicator taxa in Fig. 4, Cl. 3, and 12 indicator GO terms in Fig. 5, Cl. 3) were not considered in downstream discussion.

The 33 stable taxonomic indicators from cluster 1 collectively showed positive associations with particulate nutrients (median ± SD of MINT sPLS positive partial correlation scores for POC: 0.44 ± 0.04; PN: 0.41 ± 0.03; and PP: 0.34 ± 0.02), Chl-*a* (0.39 ± 0.03), and DOC (0.35 ± 0.02) and negative associations with dissolved inorganic nutrients (median ± SD of MINT sPLS negative partial correlation scores for NO_3_: − 0.50 ± 0.03; NO_2_: − 0.34 ± 0.04; NH_4_: − 0.26 ± 0.03; PO_4_: − 0.46 ± 0.03; and TDP: − 0.27 ± 0.04) (Fig. 4A, Cl. 1). These microbial indicators were consistent across either three trips (LOGOCV stability score = 0.75) for 17 taxa, including members of *Synechococcales*, two *Rhodobacteraceae*, and *Rhodospirillaceae*, or two trips (LOGOCV stability score = 0.5) for 16 taxa, including two *Oceanospirillaceae*, two *Rhodospirillaceae*, and two *Burkholderiaceae* (Fig. 4b, Cl. 1). The second cluster contained 34 taxa, and while also largely composed of *Alphaproteobacteria* (four *Rhodospirillaceae*), *Gammaproteobacteria* (five *Cellvibrionales*), and *Deltaproteobacteria* similar to the first cluster, these microbes were up to a fivefold less strongly associated with particulate nutrients (POC: 0.09 ± 0.06, PN: 0.10 ± 0.06, and PP: 0.09 ± 0.05) and phosphorus (PO_4_: − 0.11 ± 0.07) compared with indicators from the first cluster, but still show positive associations with DOC (0.21 ± 0.07) (Fig. 4a, Cl. 2). The third cluster (13 taxa), predominantly composed of *Flavobacteriaceae* (12 taxa), showed two distinct subgroups. Both were positively associated with dissolved nitrogen (NH_4_: 0.12 ± 0.04, NO_2_: 0.19 ± 0.06, and NO_3_: 0.14 ± 0.09), but one cluster (Fig. 4a, Cl. 3a) positively associated with particulate nutrients (POC: 0.17 ± 0.07, PN: 0.14 ± 0.06, and PP: 0.10 ± 0.05) and negatively associated with dissolved phosphorus (PO_4_: − 0.15 ± 0.07), and the other cluster (Fig. 4a, Cl. 3b) negatively associated with particulate nutrients (POC: − 0.08 ± 0.04, PN: − 0.08 ± 0.0,; and PP: − 0.06 ± 0.06) and positively associated with dissolved phosphorus (PO_4_: 0.10 ± 0.04). Overall, these patterns indicate that particulate nutrients, dissolved N and P, were the main physico-chemical variables driving partitioning of seawater microbial communities in the surveyed offshore reefs. Most of the indicator taxa were positively associated with particulate nutrients and negatively associated with dissolved N and P, with the exception of several genera in the *Flavobacteriaceae* family that were positively associated with both particulate nutrients and dissolved N and P (Fig. 4).

The 34 microbial GO terms identified in MINT sPLS as stable (i.e., reproducible across sampling trips) indicators collectively showed positive associations with particulate nutrients (median ± SD of MINT sPLS-positive partial correlation scores for POC: 0.42 ± 0.05, PN: 0.35 ± 0.05, and PP: 0.24 ± 0.07), Chl-*a* (0.29 ± 0.05), DOC (0.30 ± 0.06), and salinity (0.34 ± 0.08) and were negatively associated with dissolved nutrients (NH_4_: − 0.18 ± 0.05, NO_2_: − 0.27 ± 0.05, NO_3_: − 0.29 ± 0.06, TDP: − 0.24 ± 0.06, and PO_4_: − 0.33 ± 0.06, see Fig. 5a, Cl. 1). These stable indicator GO terms were involved in (1) transmembrane nutrient uptake, including permease proteins PstB — phosphate transport system permease protein (LOGOCV stability = 0.5) and PstC (LOGOCV stability = 0.5) as subunits of a Pst system for phosphate transport; ion transmembrane transport — Na^+^/H^+^ antiporter subunit G (LOGOCV stability = 0.75); and assimilation of external ammonium — alanine dehydrogenase (LOGOCV stability = 1); (2) utilization of N-acetylglucosamine (N-acetylglucosamine-6-phosphate deacetylase, LOGOCV stability = 1); (3) oxidative phosphorylation, such as chain 1 of the NADH-quinone oxidoreductase (LOGOCV stability = 1), as well as synthesis of (4) fatty acids — enoyl-acyl carrier protein reductase (NADH) (LOGOCV stability = 1); and (5) vitamins — pyridoxal kinase for biosynthesis of pyridoxal phosphate, an active form of vitamin B6 (LOGOCV stability = 0.5) (Fig. 5, Cl. 1). The second cluster (Fig. 5a, Cl. 2) consisted of 37 GO terms positively associated with Phaeo, salinity, PP, and dissolved nitrogen variables and negatively associated with dissolved phosphorus and DOC (Fig. 5), while the third cluster (Fig. 5a, Cl. 3) consisted of 12 GO terms only positively associated with dissolved phosphorus (TDP) (Fig. 5). Collectively, the 34 GO terms identified as stable indicators were implicated in processes including nutrient uptake, ion transport, ammonium assimilation, oxidative phosphorylation, and synthesis of fatty acids and vitamins.

### Microbial functional genes correlate more stably to physico-chemical variables than taxonomy

**Fig. 6:**
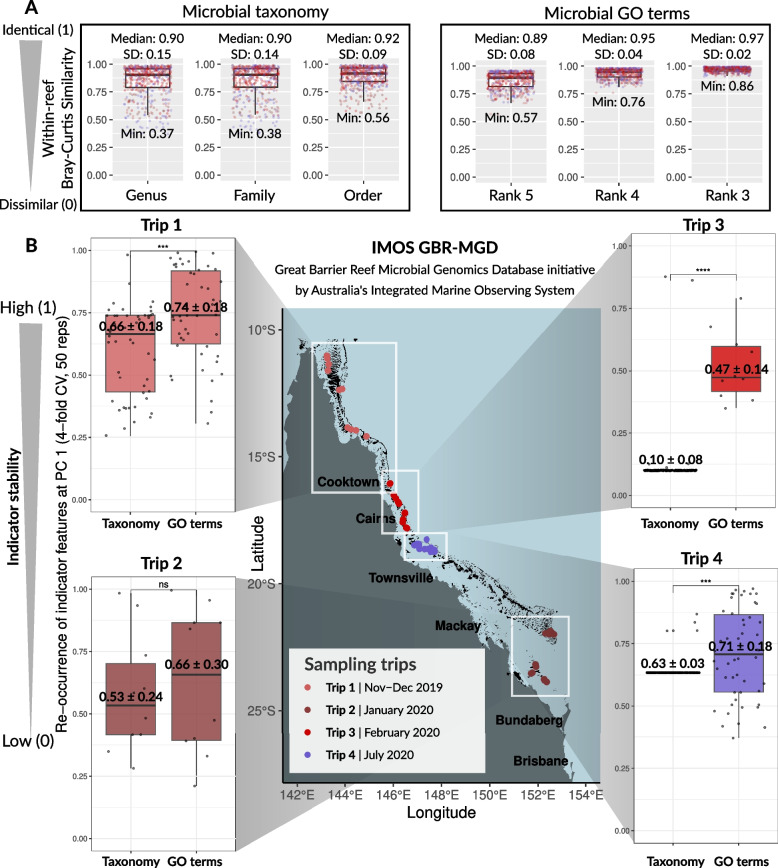
Differing diagnostic potential of microbial taxonomy and function to inform changes in continuous physico-chemical variables in the surrounding reef. Data points on the boxplots are color-coded according to the sampling trips, as indicated by the legend on the map. **A** Bray–Curtis similarity index shows within-site community similarity (0 = dissimilar; 1 = identical) for microbial taxonomy (at genus, family, and order-level classifications) and microbial functions (GO terms collapsed at ranks 5, 4, and 3). **B** Comparison of how frequently indicator microbes and indicator genes (left and right boxplots, respectively) are reselected across 200 independent sPLS cross-validation runs (fourfold CV × 50 repeats), across all four sampling trips (Trips 1–4). Higher stability scores are a proxy of robustness of the indicator signal for a corresponding microbe/gene (i.e., the stability score of 1 would mean that the indicator microbe/gene was reselected in sPLS on component 1 in each of the 200 CV runs). The symbols *, **, ***, and **** denote levels of statistical significance in pairwise Wilcoxon rank-sum tests when testing variation between stability scores from indicator taxa and GO terms within each of the four sampling trips: * for *p* < 0.05, ** for *p* < 0.01, *** for *p* < 0.001, and **** for *p* < 0.0001, indicating increasing levels of significance. “ns” indicates nonsignificant results, where *p* ≥ 0.05

To test our hypothesis that reef-associated bacterioplankton, due to functional redundancy, would exhibit higher community similarity at the functional rather than taxonomic level within a single reef site (i.e., under similar environmental conditions), we computed the Bray–Curtis similarity index (as a metric of overall compositional similarity: 0 = dissimilar, 1 = identical) between four replicates within each of the 48 surveyed reefs. This resulted in a total of 288 reef-specific Bray–Curtis similarity values (six pairwise comparisons per reef × 48 reefs) for each hierarchical level tested: for microbial taxonomy (genus, family, order, class, and phylum) and genes (GO terms at ranks 5, 4, and 3). The Bray–Curtis similarity scores for taxonomic communities showed consistent and high median values across different hierarchical levels. Specifically, the median ± SD Bray–Curtis similarity scores were as follows: 0.9 ± 0.15 at the genus level, 0.9 ± 0.14 at the family level, 0.92 ± 0.09 at the order level (Fig. 6a, microbial taxonomy), 0.93 ± 0.09 at the class level, and 0.93 ± 0.08 at the phylum level (Fig. S9). These results indicate high within-site taxonomic similarity for most of the surveyed offshore reefs. The lowest observed similarity scores were 0.37 (genus level) and 0.38 (family level) indicating that replicates within some reefs can be dissimilar at the lower taxonomic levels, although minimum similarity remains higher at higher taxonomic levels (0.56 at order-level and 0.57 at class- and phylum-level communities) (Fig. 6a, microbial taxonomy; Fig. S9). Gene profiles for reef bacterioplankton communities showed comparable median similarity scores to taxonomic communities, although with lower SD (median ± SD Bray–Curtis similarity for GO terms at rank 5: 0.90 ± 0.08, rank 4: 0.95 ± 0.04, and rank 3: 0.97 ± 0.02) and higher minimum similarity scores (0.57, 0.76, and 0.86 for GO terms collapsed at ranks 5, 4, and 3, respectively) (Fig. 6a, microbial function). Overall, replicates within a single reef site are similar both at taxonomic and functional gene levels, though this similarity is increased for functional traits.

To compare whether seawater indicator GO terms or indicator microbes have a higher stability to infer continuous physico-chemical variables in the outer GBR reefs, we generated eight sPLS models (computed for four trips × two datasets, for microbial taxa and GO terms) and perturbed them with a fourfold cross-validation repeated 50 times, resulting in 200 independent CV runs for each sPLS model. In this, we introduced a measure of statistical stability [45, 74, 75] calculated as the averaged reoccurrence of microbial indicators (taxa and GO terms, selected on sPLS dimension 1) across 200 sPLS CV runs, and the stability scores ranged from 0 (low indicator stability) to 1 (high stability). In each of the four trips, the same microbial genes/functions were more frequently reselected as indicators of physico-chemical variables compared with microbial taxa, with stability scores for indicator GO terms consistently higher (median ± SD stability for the 50 indicator GO terms on sPLS dimension 1: Trip 1 = 0.74 ± 0.18, Trip 2 = 0.66 ± 0.30, Trip 3 = 0.47 ± 0.14, and Trip 4 = 0.71 ± 0.18) compared with indicator microbes (median ± SD stability for the 50 indicator microbes on sPLS dimension 1: Trip 1 = 0.66 ± 0.18, Trip 2 = 0.53 ± 0.24, Trip 3 = 0.10 ± 0.08, and Trip 4 = 0.63 ± 0.03) (Fig. 6b, Trips 1–4). Pairwise Wilcoxon rank-sum tests confirm these trends were significant (*p* adjusted < 0.05) for Trips 1, 3, and 4, but the results were not significant in Trip 2 (*p* adjusted > 0.05, Wilcoxon rank-sum test) (Fig. 6b, Trips 1–4). Overall, these results suggest that microbial genes/function is more robustly associated with physico-chemical variables compared to microbial taxonomy.

## Discussion

The composition of reef-associated bacterioplankton undergoes significant shifts in response to environmental stressors and poor reef health conditions (reviewed in [8, 9, 14, 15]). Numerous opportunistic seawater microbes, such as *Flavobacteriaceae*, *Rhodobacteraceae* and *Vibrionaceae, *which increase in abundance during disturbances, along with their functions (e.g., virulence factors, toxin production, antibiotic resistance), have been proposed as candidate indicators of poor reef health [16, 17, 19, 37]. However, analysis efforts are lacking to evaluate if reef-associated seawater microbial taxa or genes/functions have a higher diagnostic potential in microbial monitoring and to determine whether seawater biomarkers will consistently be indicative of a specific physico-chemical metric across broad spatiotemporal scales. By employing integrative omics approaches, specifically P-integration (*sensu* [45, 69, 75]), and introducing the measure of statistical stability (i.e., reoccurrence of microbial indicators across independent cross-validation runs) into microbiome-environment associations, here we identify microbial markers stably associated with nutrient concentration across reefs and season in offshore GBR surface waters. We also show that a greater proportion of variance in gene content was attributable to physico-chemical variables compared to taxonomic composition, with functional genes/environment associations being more than twice as stable.

### Deriving seawater microbial indicators for GBR reef health monitoring

Functional redundancy proposes that environmental filtering primarily selects for functional traits in pelagic microbes [24, 25, 28, 76]. Computing reef-specific Bray–Curtis similarity scores (at various levels for microbial taxonomy and GO terms) as a metric of overall community similarity, we show that across the surveyed reefs, reef-associated bacterioplankton exhibit higher community similarity at the functional rather than taxonomic level within a single reef site, where similar environmental conditions prevail. As the observed patterns may include core genes encoding for essential functions that are critical to life and thus shared across diverse taxa [77], we further explored the robustness of these findings by focusing only on the stability of indicator microbial taxa and GO terms associated with specific physico-chemical variables in the reef environment, using sPLS analysis complemented with cross-validation. The sPLS stability scores for indicator microbial genes/functions were approximately twice as high as those for microbial taxa consistently across different regions and seasons on the GBR, further highlighting that indicator gene targets offer greater precision in monitoring environmental metrics within reef ecosystems.

These observations are consistent with the concept of functional redundancy in pelagic microbial communities where multiple members of the community possess overlapping metabolic capabilities and are able to functionally replace one another [23, 24, 28, 36]. For example, an analysis of N cycling seawater microbial communities using data from the Tara Oceans expedition reported 30.1% of variance in the composition of functional traits statistically attributed to environmental measures compared with 16.3% of variance in taxonomic composition. Further, stochastic (i.e., random) processes had ~ 1.4-fold increase in relative importance of shaping the taxonomic compared to functional compositional variance, suggesting N-cycling microbial functions are more influenced by deterministic processes (i.e., environmental filtering) compared to taxonomy [25]. This explains why genes encoding for the same N-cycling pathways were consistently enriched in the epipelagic (N_2_ fixation, organic decomposition, and assimilatory nitrite reduction to ammonia) and mesopelagic (nitrification, dissimilatory nitrate reduction to nitrite, and annamox) zones, whereas the taxonomic composition of N-cycling microbes between depth layers varied substantially, even at phylum level [25]. Taken together, the findings indicate that functional traits in seawater microbial communities are tightly linked to environmental measures and thus more likely to reflect the environment than taxonomy does. Functional redundancy may broadly contribute to ecosystem resilience against perturbations [23, 76, 78–80]. Since resilience is a key measure in ecosystem monitoring and management [81], we posit that gene content could conceivably serve as an indicator of ecosystem resilience, and changes in gene content coupled with contextual metadata could better reveal insight into the state of reef ecosystems compared with taxonomic indicators.

### *Synechococcus* and *Prochlorococcus* are central to the production of particulate nutrients

**Fig. 7:**
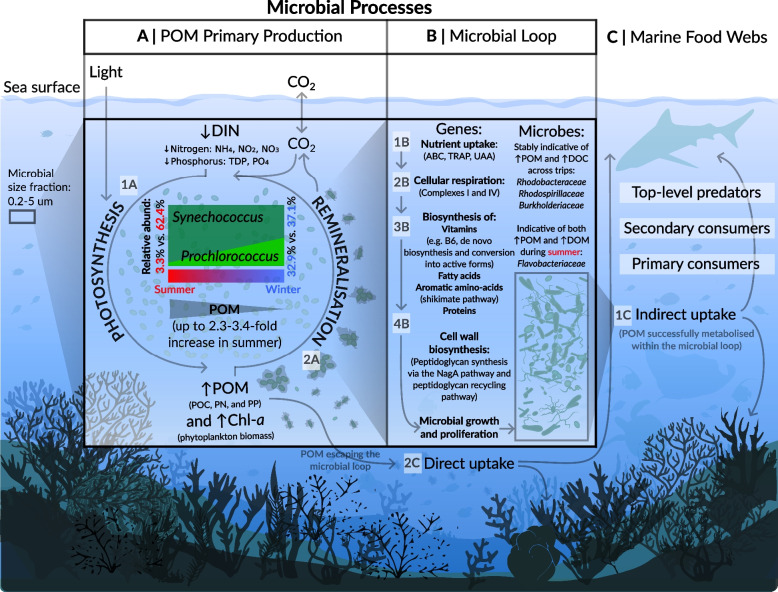
Conceptual overview summarizing the roles of seawater microbiomes in nutrient cycling in offshore GBR surface waters. The planktonic picocyanobacteria *Synechococcus* and *Prochlorococcus* play key roles in nutrient cycling: they uptake dissolved inorganic nutrients (DIN) such as nitrogen (ammonium — NH_4_, nitrite — NO_2_, and nitrate — NO_3_) and phosphorus (phosphate — PO_4_), reducing DIN concentrations (1A). In the presence of light and carbon dioxide (CO_2_), the uptaken DIN will be used during photosynthesis to produce particulate organic matter (POM) including organic carbon (POC), nitrogen (PN), and phosphorus (PP), overall resulting in elevated POM concentrations and higher biomass of these picocyanobacteria, indicated via elevated chlorophyll a (Chl-*a*) (1A). During summer, elevated photosynthesis rates primarily by *Synechococcus* result in up to a threefold increase in POM production, whereas during winter, nutrient concentrations are lower, and we also observe notable contributions of *Prochlorococcus* to POM production (1A). A fraction of POM deriving from *Synechococcus* and *Prochlorococcus* will be remineralised (2A) by (**B)** entering the microbial loop. Here, a diverse consortium of seawater heterotrophic microbes, notably *Rhodobacteraceae*, *Rhodospirillaceae*, *Oceanospirillaceae*, *Burkholderiaceae* and *Flavobacteriaceae*, will benefit from nutrient-rich conditions by encoding genes for (1B) nutrient uptake and (2B) cellular respiration to generate energy, which can be directed towards (3B) synthesis of complex compounds and (4B) microbial growth. As a result of microbial activity on phytoplankton-derived POM, organic molecules originally present in particulate form are remineralized into DIN (NH₄, NO_2_, NO₃, PO₄) and dissolved organic carbon (DOC). These dissolved nutrients are then available for uptake by other organisms, including *Synechococcus* and *Prochlorococcus* which can photosynthesize again (1A), ultimately recycling POM in offshore GBR waters and making it available to higher trophic levels (**C**). POM from these picocyanobacteria may enter marine food webs via two pathways: 1C an indirect pathway, where heterotrophic seawater microbes that successfully integrated phytoplankton-derived POM into their biomass will be grazed by flagellates and microzooplankton, which in turn will support larger macroorganisms; or 2C through direct uptake of POM that escapes immediate metabolism by heterotrophic seawater microbes, thus bypassing the microbial loop

The chemistry of GBR surface waters is characterized by a cluster of five collinear physico-chemical variables (Chl-*a*, Phaeo, POC, PN, and PP) and a weak collinearity of dissolved nutrients (DOC, NH_4_, NO_2_, NO_3_, PO_4_, and Si) consistently elevated by 10–50% during summer and with the lowest nutrient concentrations typically observed during winter and early spring in August–September [38, 82, 83]. Largely consistent with published data, we observed that temperature and nutrient concentrations were consistently higher in austral summer than in winter, apart from TDP and PO_4_ which were higher in austral winter potentially due to seasonal upwelling of nutrient-rich water from the Coral Sea into reefs on the outer continental shelf, via intrusive upwelling events that are documented to occur in the central GBR [84, 85]. We also observed collinearity between particulate nutrients (POC, PN, and PP) and Chl-*a* (proxy of phytoplankton biomass), indicating that particulate nutrients (≥ 0.7 µm in diameter) in the studied microbial size fraction (0.2–5 µm) may originate from the picoplankton biomass (Fig. 7a), most likely from picocyanobacteria *Synechococcus* (~ 1 µm) and *Prochlorococcus* (~ 0.5 µm) which cumulatively comprised 66.92% of annotated sequences in our data. *Synechococcus* and *Prochlorococcus* usually dominate phytoplankton biomass in GBR waters [19, 84, 86, 87], benefiting from favorable light conditions in offshore GBR reefs that facilitate photosynthesis. Therefore, particulate organic matter (POM) in the outer GBR predominantly originates from marine phytoplankton [88], contrasting with the terrestrial origin of POM found in riverine zones, inner estuarine mixing zones, and inshore reefs, with minimal amounts reaching the outer GBR [88]. Our results further suggest that POM in the outer GBR is predominantly produced by *Synechococcus* during summer (average 62.38% and 3.23% relative abundance in summer trips for *Synechococcus* and *Prochlorococcus*, respectively), whereas during winter, we also observe considerable contribution of *Prochlorococcus* to POM production (average 37.02% and 32.93% relative abundance in the winter trip for *Synechococcus* and *Prochlorococcus*, respectively) (Fig. 7a). These picocyanobacteria have relevance to prospective monitoring since an increasing *Synechococcus*-*Prochlorococcus* abundance ratio was proposed as an index for elevated cross-shelf nutrient loads in reef waters [19], and we posit extending this index to a wider swath of offshore reefs, with *Synechococcus* indicative of high particulate nutrient loads broadly across the GBR. To further validate our proposed model, it would be beneficial to incorporate cell count data for *Synechococcus* and *Prochlorococcus* in future sampling efforts, as well as consider benthic cover organisms since emerging evidence suggests that corals exhibit preferential feeding on *Synechococcus*, potentially affecting their abundances [89, 90]. Such approaches will enhance our understanding of picocyanobacterial contributions to POM dynamics and nutrient cycling in reef ecosystems.

Negative correlations were observed between particulate nutrients (POC, PN, PP) and Chl-*a* with dissolved inorganic nutrients (NH_4_, NO_2_, NO_3_, PO_4_, and TDP) (Fig. 4a), likely because this production of phytoplankton-derived POM from newly fixed carbon requires the uptake and assimilation of dissolved nutrients such as N, P, and trace metals [86]. Dissolved inorganic nutrients in shelf waters are rapidly taken up by growing phytoplankton (i.e., ~ 8–24 h for dissolved nitrogen and ~ 24 h for dissolved phosphorus, see e.g. [86, 91, 92]) including *Synechococcus* and *Prochlorococcus*, which are highly efficient at using dissolved nutrients and exhibit a capacity for near-maximal growth down to available DIN levels of ≤ 0.02 µM, concentrations similar to the minimum detection levels [86]. Based on our findings and existing literature, we propose a mechanistic explanation, whereby picocyanobacteria *Synechococcus* and *Prochlorococcus* initially uptake dissolved nitrogen and phosphorus (resulting in decreased DIN concentrations), subsequently producing POM (POC, PN, and PP) during photosynthesis (Fig. 7, 1a). This process leads to increased phytoplankton biomass, as indicated by elevated Chl-*a*, and ultimately results in the observed collinear relationship between Chl-*a* and POM, negatively correlating with the uptake of dissolved inorganic nutrients (Fig. 4, Fig. 5, Fig. 7).

Several GO terms identified in our analysis are potentially involved in the uptake of dissolved nitrogen and phosphorus. For instance, alanine dehydrogenase, indicative of low ammonium concentrations across all sampling trips, likely plays a role in ammonium assimilation by catalyzing the synthesis of L-alanine from pyruvate and external ammonium [93]. Additionally, two subunits of the phosphate transport system (Pts), PstB (the catalytic subunit) and PstC (the transmembrane portion), were consistently enriched in low phosphate environments across sampling trips, suggesting an adaptive response to increase the uptake of limited inorganic phosphate [94]. While these GO terms positively correlated to Chl-*a* (proxy for phytoplankton biomass), further research is necessary to attribute these genes/functions to *Synechococcus* and *Prochlorococcus*, lineages well-documented for their genomic heterogeneity, making genomic reconstructions from environmental metagenomics problematic [95–99]. Lastly, we also observe strong collinearity between phytoplankton-derived POM with DOC, and while DOC can be a product of extracellular release from actively photosynthesizing phytoplankton [100–102], alternative microbial processes may also produce DOC [103], including (1) senescing and dead phytoplankton [104, 105], (2) sloppy feeding during zooplankton grazing [101, 102, 106, 107], (3) POM dissolution by heterotrophic microbes [108–110], and (4) viral lysis [42]. Both DOC (regardless of its origin) and phytoplankton-derived POM can then enter the microbial loop [111–113] where a diverse consortium of seawater heterotrophic bacteria will benefit from nutrient-rich conditions (Fig. 7, 2b).

### Phytoplankton-derived nutrients fuel the microbial loop and support higher trophic levels

Free-living pelagic microorganisms surrounding coral reefs enable the capture, retention, and recycling of nutrients and trace elements, essential to maintaining reef ecosystems in oligotrophic environments often likened to “nutrient deserts” [12, 114, 115]. Heterotrophic seawater microbes positively associated with elevated POM and DOC in the surveyed offshore reefs included members of *Gammaproteobacteria* (two *Oceanospirillaceae*), *Alphaproteobacteria *(three *Rhodospirillaceae*, three *Rhodobacteraceae*), and two *Burkholderiaceae* (Fig. 7b). These microbes are frequently documented as enriched under elevated nutrients within coral reefs [11, 19, 21, 38, 39]. For example, *Rhodobacteraceae* are noted for their association with dissolved nutrients in inshore GBR reefs dominated by macroalgae (as observed by [11, 19, 39]). Our findings show *Rhodobacteraceae* to be consistently enriched with elevated particulate nutrients in offshore GBR reefs, indicating their role as versatile heterotrophic marine bacteria [116] potentially capable of utilizing both dissolved and particulate nutrients in the GBR. Various members of *Rhodospirillaceae* also indicated high levels of particulate nutrients, although previous studies have reported their association with decreasing nutrient levels [19]. This discrepancy likely stems from their broad metabolic potential, which includes diazotrophic capabilities, opportunistic pathogenesis, and adaptation to various aerobic and anaerobic conditions [19, 117], ultimately allowing *Rhodospirillaceae* to adapt to various niches across reef environments. Lastly, *Flavobacteriaceae*, known for their capacity to degrade complex polysaccharides and utilize diverse carbon sources [118], were the only group in our data enriched when both particulate and dissolved nutrient concentrations were elevated. Interestingly though, the MINT sPLS LOGOCV stability scores suggest that the signal of *Flavobacteriaceae* as indicators was not consistent across trips. This instability, coupled with low MINT sPLS correlation scores, suggests that *Flavobacteriaceae* are summer-specific indicators of elevated nutrients in the offshore GBR as *Flavobacteriaceae* were the most discriminatory of summer trips in our data, increasing both in relative abundance and diversity. This is in addition to the relevance of *Flavobacteriaceae* as indicators of labile polysaccharides released from macroalgae in inshore GBR reefs (as proposed by [11, 19, 39]) where macroalgae cover is comparatively higher than in the offshore GBR.

Numerous genes encoding for nutrient uptake systems were enriched in the GBR samples when DOC and phytoplankton-derived POM are available (Fig. 7, 1b), including ABC (ATP-binding cassette) transporters, TRAP (tripartite ATP-independent periplasmic) transporter permease proteins, UAA (uncharacterized amino acid) transporters, and various ion transporters. Concurrently, we found an enrichment of microbial Gene Ontology (GO) terms related to energy metabolism and cellular respiration (Fig. 7, 2b), such as NADH-quinone oxidoreductase (IPR010226, complex I of the respiratory chain) and cytochrome c oxidase subunit III (IPR024791, subunit of the terminal complex IV in the respiratory chain). These gene pathways drive electron transport and are coupled to proton transmembrane transport, generating a proton motive force for ATP synthesis, ultimately contributing to increased energy metabolism [119–122]. The energy generated from nutrient uptake and cellular respiration can then be directed towards anabolic metabolism and synthesis of various complex compounds [123–125], and we observed high representation of gene pathways involved in biosynthesis of vitamins, fatty acids, amino acids, and proteins (Fig. 7, 3b). For example, vitamin B6 biosynthesis appears widespread in GBR bacterioplankton, facilitated by the 4-hydroxythreonine-4-phosphate dehydrogenase PdxA (IPR005255) [126] and pyridoxal kinase enzymes (IPR004625) [127–129], which were consistently enriched in samples from at least two trips (LOGOCV stability = 0.5), indicating that elevated particulate nutrients promote this biosynthesis, as also observed in pelagic microbes [125]. Further, the consistent presence of genes associated with fatty acid and amino acid biosynthesis indicates that elevated DOC and phytoplankton-derived POM in the GBR supports increased synthesis of these compounds. Specifically for fatty acid biosynthesis, NADH-dependent enoyl-acyl carrier protein reductase (IPR014358) was stably indicative of elevated nutrients in each sampling trip (LOGOCV stability score = 1), facilitating fatty acid biosynthesis by reducing the enoyl-acyl carrier protein (ACP) intermediate to produce saturated acyl-ACP [130, 131]. For amino-acid biosynthesis, chorismate synthase (IPR000453), catalyzing the final step of the shikimate pathway used by prokaryotes to synthesize aromatic amino acids [132], was stably (i.e., in three sampling trips, LOGOCV stability = 0.75) enriched with elevated DOC and phytoplankton-derived POM, suggesting enhanced amino acid biosynthesis in reef bacterioplankton under nutrient-rich conditions. As building blocks of proteins, these amino-acids are likely used in subsequent protein synthesis since various ribosomal proteins as essential components of protein-translation organelles ribosomes [133] were indicative of elevated POM and DOC. The indicator ribosomal proteins were as follows: S6 (IPR000529) and L16 (IPR000114) identified as stable indicators across three and two sampling trips (respectively), the trip-specific L5 (IPR002132, IPR020930), and S5 (IPR000851, IPR005712) (Fig. 5, Fig. 7, 3b).

Enhanced biosynthesis of complex compounds in nutrient-rich conditions can support the cellular growth and proliferation of reef-associated heterotrophic seawater microbes (Fig. 7, 4b). Crucial to the bacterial cell cycle (elongation and division) is the synthesis of bacterial cell walls which consist of peptidoglycans, composed of alternating units of N-acetylglucosamine (NAG) and N-acetylmuramic acid (NAM) connected via the β-(1,4)-glycosidic bond [134, 135]. The NagA gene (N-acetylglucosamine-6-phosphate deacetylase — IPR003764) was persistently indicative of elevated DOC and phytoplankton-derived POM in the offshore GBR (i.e., in each of the four sampling trips), potentially facilitating the NAG breakdown to produce glucosamine-6-phosphate for synthesis of bacterial cell walls via the peptidoglycan recycling pathway [136]. NAG are among the largest pools of amino sugars in the ocean [137], and NAG utilization is consistent with a previous metagenomic study in inshore reefs of the central GBR, where NAG transporters were identified in reef water microbes, though absent from sponge and macroalgae microbiomes [39]. Further, anhydro-N-acetylmuramic acid kinase (IPR005338) was also enriched under elevated POM and DOC, another enzyme crucial for peptidoglycan recycling through phosphorylation of the anhydro-N-acetylmuramic acid (anhMurNAc) to produce MurNAc-6-phosphate, an intermediate in peptidoglycan metabolism during cell wall remodelling and turnover [138, 139]. Consistent enrichment of these two enzymes across the GBR highlights that both bacterial cell wall biosynthesis and maintenance (indicative of microbial growth and proliferation) are widespread in heterotrophic seawater microbes when DOC and phytoplankton-derived POM are available (Fig. 7, 4b). Further investigation into the metabolic activities of these indicator microbes and genes, using techniques such as metatranscriptomic and metaproteomic analyses, as well as stable isotopes, could provide richer insights into how nutrient availability influences the composition and metabolism of GBR seawater microbial communities.

Five physico-chemical variables, including salinity, total suspended solids (TSS), total dissolved nitrogen (TDN), silica (Si), and nitrate (NO_3_), did not significantly influence the overall community composition or functional potential (Fig. 2c). This is likely due to our sampling design where all sites are offshore reefs, and therefore, some metrics have a low explanatory value as they are highly consistent across this longitudinal gradient. Salinity, for example, has been well-documented as one of primary factors shaping community composition in aquatic microbes [71] and was reported to explain 4.2% of community variation (according to variation partitioning analysis) in the GBR seawater microbiomes [19]. However, inshore sites influenced by freshwater input and therefore a stronger salinity gradient were investigated in [19], while our data captured a low degree of variation in salinity (34.6 to 35.8 practical salinity units — PSU, Table 1), which is likely why salinity was not significant in our study (Fig. 2c). Moving forward, reevaluation of these specific physico-chemical variables should occur in the future if additional sampling introduces a broader range of sites, particularly areas of inshore reefs, where proximity to land and human activities contributes to a wider range of environmental variation.

## Conclusions

Our study provides a functional baseline for reef-associated bacterioplankton across offshore regions of the GBR, demonstrating that microbial functional genes have a higher stability than taxonomy in inferring physico-chemical variables across broad spatiotemporal scales. When dissolved organic carbon (DOC) and phytoplankton-derived particulate organic matter (POM) are elevated in offshore GBR reefs, microbial genes and functions we found as consistently enriched in heterotrophic seawater microbes collectively point towards enhanced microbial nutrient uptake (Fig. 7, 1b) and energy generation through cellular respiration (Fig. 7, 2b), supporting anabolic metabolism and synthesis of complex compounds (Fig. 7, 3b) to ultimately increase growth and biomass of heterotrophic seawater microbes (Fig. 7, 4b). Members of reef bacterioplankton that increased in relative abundances with elevated POM and DOC consistently across seasons/sectors in the offshore GBR included *Rhodospirillaceae*, *Rhodobacteraceae*, and *Burkholderiaceae*, whereas *Flavobacteriaceae* were enriched when both dissolved and particulate nutrients were elevated, although predominantly during summer (Fig. 7b). These heterotrophic marine microorganisms can then be grazed by flagellates and microzooplankton which in turn support larger macroorganisms, ultimately transferring nutrients derived from *Synechococcus* and *Prochlorococcus* to higher trophic levels in offshore GBR reefs (Fig. 7, 1c). Phytoplankton-derived POM (i.e., retained on a filter with a pore size of approximately 0.7 μm) not immediately metabolized by heterotrophic seawater microbes will escape the microbial loop, also becoming available to benthic and pelagic organisms at higher trophic levels through direct uptake (Fig. 7, 2c). In summary, *Synechococcus* and *Prochlorococcus* are crucial components of the marine food web in offshore regions of the GBR, supporting various levels of the ecosystem through their role as primary producers and their contributions to nutrient cycling and carbon sequestration.

Since microbial genes had higher indicator stability scores and functional redundancy is a well-established phenomenon for pelagic microbes [23–25, 30], we assert that microbial functions have a higher utility than microbial taxa for rapid assessment of reef ecosystem health. It is worth noting, however, that this study was conducted using taxonomic annotations derived from metagenomic reads, which may provide less resolution than 16S-based taxonomic annotations. Microbial transcriptomic profiling assays and biosensors, already used in environmental toxicity testing [140] to detect heavy metal pollution [141] and track hydrocarbon degradation from oil spills [142], would benefit from improved collaboration between researchers and reef managers to identify the most suitable microbial markers (taxa or genes/functions) for developing targeted microbial-based assays for rapid reef health assessment. Lastly, as reef metagenomes become more widely available [14, 37, 143], it will be possible to cross-examine datasets across global scales and integrate microbial responses to generate spatiotemporally coherent baselines of microbes indicating reef health; however, care will be needed to distinguish microbial biomarkers from confounding factors such as geography and season. To complement these emerging large-scale surveys of reef seawater microbes, it will be crucial to capture the state of reef bacterioplankton over time as is being recorded for pelagic microbes [144, 145], for example, at (1) long-term ocean time-series stations (which are yet to be established, unlike the 72 microbial observatories catalogued so far for pelagic microbes, see [146]), at (2) day-to-day resolution [147, 148] and across (3) mesoscale processes [149]. Such baselines of reef-associated (bacterio)plankton will be invaluable in facilitating identification of deviations that could signal impending disturbance events [19, 150] and link how microbial community shifts contribute to ecosystem stability and transition to alternative stable states.

## Supplementary Information

Supplementary Material 1.

## Data Availability

Raw sequencing data and the associated physico-chemical variables have been uploaded to the IMOS-AODN repository and are available at: Australian Institute of Marine Science (AIMS). (2022). Great Barrier Reef Genomics Database: Seawater Illumina Reads. https://doi.org/10.25845/Q4XH-YN10. The code to replicate the analysis is available at: https://github.com/mterzin/IMOS_GBR_MGD_read-centric_analysis.

## Abbreviations

ABC: ATP-binding cassette
AIMS: Australian Institute of Marine Science
Chl-*a*: Chlorophyll a
CLR: Center log ratio
DIAMOND: Alignment tool
DOC: Dissolved organic carbon
ECO-FLNTU-RT: Fluorescent and turbidity sensor (real time)
FastQC: Quality control software
GBR: Great Barrier Reef
GO: Gene Ontology
IMOS: Integrated Marine Observing System
Inkscape: Vector graphics software
IPR: InterPro
LOGOCV: Leave-one-group-out cross-validation
MEGAN: Metagenome Analyzer
Millipore Sterivex-GP: Pressure Filter
MINT: Multivariate INTegrative method
MINT sPLS: Multivariate INTegration Sparse Partial Least Squares
N: Nitrogen
NADH: Nicotinamide adenine dinucleotide (NAD) + hydrogen (H)
NAG: N-Acetylglucosamine
NAM: N-Acetylmuramic acid
NanoDrop: Spectrophotometer
NH_4_: Ammonium
NO_2_: Nitrite
NO_3_: Nitrate
NovaSeq: Sequencing System
PCA: Principal Component Analysis
PERMANOVA: Permutational multivariate analysis of variance
Phaeo: Phaeophytin
PLS: Partial least squares
PO_4_: Phosphate
POC: Particulate organic carbon
PN: Particulate nitrogen
PP: Particulate phosphorus
Qubit: Fluorometer for DNA quantification
R: Programming language
RV: Research vessel
Sartorius: Filter manufacturer
Sartorius Minisart N: Syringe filter
SBE: Sea-Bird Electronics
Shimadzu TOC-L: Carbon analyzer
Si: Silicate
sPLS: Sparse partial least squares
SST: Sea surface temperature
TARA: Tara Oceans Foundation
TDN: Total dissolved nitrogen
TDP: Total dissolved phosphorus
TRAP: Tripartite ATP-independent periplasmic
Trimmomatic: Quality-filtering software
TSS: Total suspended solids
UAA: Uncharacterized amino acid
VEGAN: R package for diversity analysis
µg: Microgram,
µm: Micrometer
mm: Millimeter
cm: Centimeter
h: Hour
L: Liter
