# Supplementary material for: Gene content of seawater microbes is a strong predictor of water chemistry across the Great Barrier Reef
Source: Microbiome. 2025 Jan 16;13:11. doi: 10.1186/s40168-024-01972-0 (PMC11737092; doi:10.1186/s40168-024-01972-0)
Supplement: Supplementary file 2 — Supplementary Material 1. [file 40168_2024_1972_MOESM1_ESM.pdf]

Supplementary Material

Table S1. Statistics on Illumina sequencing before and after quality filtering.

Sample_ID	Raw_counts	After_Trimmomatic	Percentage retained
11-049-1_S89_R1	11391661	9162136	80.43
11-049-1_S89_R2	11391661	9162136	80.43
11-049-2_S90_R1	9506774	7178113	75.51
11-049-2_S90_R2	9506774	7178113	75.51
11-049-3_S91_R1	19387690	15648432	80.71
11-049-3_S91_R2	19387690	15648432	80.71
11-049-4_S92_R1	19484249	15795415	81.07
11-049-4_S92_R2	19484249	15795415	81.07
11-162-1_S81_R1	16545320	12753615	77.08
11-162-1_S81_R2	16545320	12753615	77.08
11-162-2_S82_R1	14615572	10176877	69.63
11-162-2_S82_R2	14615572	10176877	69.63
11-162-3_S83_R1	19143379	14946146	78.07
11-162-3_S83_R2	19143379	14946146	78.07
11-162-4_S84_R1	22970532	17895321	77.91
11-162-4_S84_R2	22970532	17895321	77.91
13-124-1_S9_R1	19915667	15869540	79.68
13-124-1_S9_R2	19915667	15869540	79.68
13-124-2_S10_R1	22747351	16339989	71.83
13-124-2_S10_R2	22747351	16339989	71.83
13-124-3_S11_R1	16804263	13278069	79.02
13-124-3_S11_R2	16804263	13278069	79.02
13-124-4_S12_R1	21950573	17036503	77.61
13-124-4_S12_R2	21950573	17036503	77.61
21-550-1_S69_R1	20100727	16218922	80.69
21-550-1_S69_R2	20100727	16218922	80.69
21-550-2_S70_R1	22553087	18460291	81.85
21-550-2_S70_R2	22553087	18460291	81.85
21-550-3_S71_R1	21563329	16412859	76.11
21-550-3_S71_R2	21563329	16412859	76.11
21-550-4_S72_R1	26678391	20829016	78.07
21-550-4_S72_R2	26678391	20829016	78.07
21-580-1_S57_R1	21996050	17534464	79.72
21-580-1_S57_R2	21996050	17534464	79.72
21-580-2_S58_R1	17510809	14002231	79.96
21-580-2_S58_R2	17510809	14002231	79.96
21-580-3_S59_R1	21164806	16686850	78.84
21-580-3_S59_R2	21164806	16686850	78.84
21-580-4_S60_R1	22786331	18154819	79.67
21-580-4_S60_R2	22786331	18154819	79.67
22-084-1_S41_R1	21554612	17317715	80.34
22-084-1_S41_R2	21554612	17317715	80.34
22-084-2_S42_R1	23120185	18544566	80.21
22-084-2_S42_R2	23120185	18544566	80.21
22-084-3_S43_R1	20633510	16549809	80.21
22-084-3_S43_R2	20633510	16549809	80.21
22-084-4_S44_R1	18036072	13208697	73.23
22-084-4_S44_R2	18036072	13208697	73.23
Agincourt1-1_S33_R1	18208575	14678322	80.61

Agincourt1-1_S33_R2	18208575	14678322	80.61
Agincourt1-2_S34_R1	14998998	11433509	76.23
Agincourt1-2_S34_R2	14998998	11433509	76.23
Agincourt1-3_S35_R1	15827149	13030639	82.33
Agincourt1-3_S35_R2	15827149	13030639	82.33
Agincourt1-4_S36_R1	16259240	13367259	82.21
Agincourt1-4_S36_R2	16259240	13367259	82.21
Arlington-1_S37_R1	12936835	10580761	81.79
Arlington-1_S37_R2	12936835	10580761	81.79
Arlington-2_S38_R1	12186989	9966004	81.78
Arlington-2_S38_R2	12186989	9966004	81.78
Arlington-3_S39_R1	12372043	9706692	78.46
Arlington-3_S39_R2	12372043	9706692	78.46
Arlington-4_S40_R1	20072805	16473992	82.07
Arlington-4_S40_R2	20072805	16473992	82.07
Boult-1_S25_R1	21121009	16974878	80.37
Boult-1_S25_R2	21121009	16974878	80.37
Boult-2_S26_R1	22444634	18484731	82.36
Boult-2_S26_R2	22444634	18484731	82.36
Boult-3_S27_R1	16841533	13434588	79.77
Boult-3_S27_R2	16841533	13434588	79.77
Boult-4_S28_R1	18856352	15363196	81.47
Boult-4_S28_R2	18856352	15363196	81.47
Broomfield-1_S49_R1	18982594	15580233	82.08
Broomfield-1_S49_R2	18982594	15580233	82.08
Broomfield-2_S50_R1	48579	3767	7.75
Broomfield-2_S50_R2	48579	3767	7.75
Broomfield-3_S51_R1	19790260	16241262	82.07
Broomfield-3_S51_R2	19790260	16241262	82.07
Broomfield-4_S52_R1	24245268	19962172	82.33
Broomfield-4_S52_R2	24245268	19962172	82.33
Broomfield-rpt-2_S115_R1	16677916	13077884	78.41
Broomfield-rpt-2_S115_R2	16677916	13077884	78.41
Centipede-1_S57_R1	14292259	10763055	75.31
Centipede-1_S57_R2	14292259	10763055	75.31
Centipede-2_S58_R1	15073864	12048345	79.93
Centipede-2_S58_R2	15073864	12048345	79.93
Centipede-3_S59_R1	13760054	11090867	80.6
Centipede-3_S59_R2	13760054	11090867	80.6
Centipede-4_S60_R1	14408385	11474288	79.64
Centipede-4_S60_R2	14408385	11474288	79.64
Chicken-1_S69_R1	13205982	10405605	78.79
Chicken-1_S69_R2	13205982	10405605	78.79
Chicken-2_S70_R1	17214103	13766095	79.97
Chicken-2_S70_R2	17214103	13766095	79.97
Chicken-3_S71_R1	13245279	10339532	78.06
Chicken-3_S71_R2	13245279	10339532	78.06
Chicken-4_S72_R1	15847423	12473351	78.71
Chicken-4_S72_R2	15847423	12473351	78.71
Chinaman-1_S65_R1	19371128	15913263	82.15
Chinaman-1_S65_R2	19371128	15913263	82.15
Chinaman-2_S66_R1	21106100	16757447	79.4
Chinaman-2_S66_R2	21106100	16757447	79.4

Chinaman-3_S67_R1	19379451	15128925	78.07
Chinaman-3_S67_R2	19379451	15128925	78.07
Chinaman-4_S68_R1	21248990	16928948	79.67
Chinaman-4_S68_R2	21248990	16928948	79.67
Corbett-1_S17_R1	14621387	11474069	78.47
Corbett-1_S17_R2	14621387	11474069	78.47
Corbett-2_S18_R1	20871896	16470187	78.91
Corbett-2_S18_R2	20871896	16470187	78.91
Corbett-3_S19_R1	22095113	16567974	74.98
Corbett-3_S19_R2	22095113	16567974	74.98
Corbett-4_S20_R1	19415512	15233388	78.46
Corbett-4_S20_R2	19415512	15233388	78.46
Davie-1_S1_R1	22015462	17908923	81.35
Davie-1_S1_R2	22015462	17908923	81.35
Davie-2_S2_R1	19501345	15511415	79.54
Davie-2_S2_R2	19501345	15511415	79.54
Davie-3_S3_R1	20188921	16126387	79.88
Davie-3_S3_R2	20188921	16126387	79.88
Davie-4_S4_R1	12946457	10219690	78.94
Davie-4_S4_R2	12946457	10219690	78.94
Erskine-1_S61_R1	14284277	10848583	75.95
Erskine-1_S61_R2	14284277	10848583	75.95
Erskine-2_S62_R1	13439806	10151986	75.54
Erskine-2_S62_R2	13439806	10151986	75.54
Erskine-3_S63_R1	15193999	11527881	75.87
Erskine-3_S63_R2	15193999	11527881	75.87
Erskine-4_S64_R1	16105531	12841271	79.73
Erskine-4_S64_R2	16105531	12841271	79.73
Fairfax-1_S33_R1	27456473	21098696	76.84
Fairfax-1_S33_R2	27456473	21098696	76.84
Fairfax-2_S34_R1	23861979	18895715	79.19
Fairfax-2_S34_R2	23861979	18895715	79.19
Fairfax-3_S35_R1	22929934	17993982	78.47
Fairfax-3_S35_R2	22929934	17993982	78.47
Fairfax-4_S36_R1	22162467	18334455	82.73
Fairfax-4_S36_R2	22162467	18334455	82.73
Farquaharson-1_S1_R1	14635499	11660269	79.67
Farquaharson-1_S1_R2	14635499	11660269	79.67
Farquaharson-2_S2_R1	15345357	11844876	77.19
Farquaharson-2_S2_R2	15345357	11844876	77.19
Farquaharson-3_S3_R1	12975351	10519862	81.08
Farquaharson-3_S3_R2	12975351	10519862	81.08
Farquaharson-4_S4_R1	13121583	10593460	80.73
Farquaharson-4_S4_R2	13121583	10593460	80.73
Feather-1_S5_R1	10834100	8679881	80.12
Feather-1_S5_R2	10834100	8679881	80.12
Feather-2_S6_R1	17316450	13332799	76.99
Feather-2_S6_R2	17316450	13332799	76.99
Feather-3_S7_R1	12855246	10216539	79.47
Feather-3_S7_R2	12855246	10216539	79.47
Feather-4_S8_R1	11193091	8782905	78.47
Feather-4_S8_R2	11193091	8782905	78.47
Fork-1_S49_R1	13227928	10508991	79.45
Fork-1_S49_R2	13227928	10508991	79.45
Fork-2_S50_R1	14791708	11564354	78.18

Fork-2_S50_R2	14791708	11564354	78.18
Fork-3_S51_R1	12033145	9399583	78.11
Fork-3_S51_R2	12033145	9399583	78.11
Fork-4_S52_R1	11840621	9059843	76.51
Fork-4_S52_R2	11840621	9059843	76.51
Grub-1_S65_R1	16345885	12830110	78.49
Grub-1_S65_R2	16345885	12830110	78.49
Grub-2_S66_R1	12862409	9968730	77.5
Grub-2_S66_R2	12862409	9968730	77.5
Grub-3_S67_R1	16660304	12962238	77.8
Grub-3_S67_R2	16660304	12962238	77.8
Grub-4_S68_R1	15196158	11898219	78.3
Grub-4_S68_R2	15196158	11898219	78.3
Hastings-1_S41_R1	15694329	12439223	79.26
Hastings-1_S41_R2	15694329	12439223	79.26
Hastings-2_S42_R1	14203851	10859760	76.46
Hastings-2_S42_R2	14203851	10859760	76.46
Hastings-3_S43_R1	17815673	14399848	80.83
Hastings-3_S43_R2	17815673	14399848	80.83
Hastings-4_S44_R1	16618775	13580106	81.72
Hastings-4_S44_R2	16618775	13580106	81.72
Hedley-1_S21_R1	13054511	10574148	81
Hedley-1_S21_R2	13054511	10574148	81
Hedley-2_S22_R1	14054961	11098597	78.97
Hedley-2_S22_R2	14054961	11098597	78.97
Hedley-3_S23_R1	13165314	10729411	81.5
Hedley-3_S23_R2	13165314	10729411	81.5
Helix-1_S61_R1	16564330	13202851	79.71
Helix-1_S61_R2	16564330	13202851	79.71
Helix-2_S62_R1	13562187	10585635	78.05
Helix-2_S62_R2	13562187	10585635	78.05
Helix-3_S63_R1	19197802	15335565	79.88
Helix-3_S63_R2	19197802	15335565	79.88
Helix-4_S64_R1	13754412	10555498	76.74
Helix-4_S64_R2	13754412	10555498	76.74
Hoskyn-1_S29_R1	18943463	15251668	80.51
Hoskyn-1_S29_R2	18943463	15251668	80.51
Hoskyn-2_S30_R1	20376534	16323841	80.11
Hoskyn-2_S30_R2	20376534	16323841	80.11
Hoskyn-3_S31_R1	23809747	19714232	82.8
Hoskyn-3_S31_R2	23809747	19714232	82.8
Hoskyn-4_S32_R1	21466806	17025580	79.31
Hoskyn-4_S32_R2	21466806	17025580	79.31
JohnBrewer-1_S93_R1	20229504	15505862	76.65
JohnBrewer-1_S93_R2	20229504	15505862	76.65
JohnBrewer-2_S94_R1	17866582	13087586	73.25
JohnBrewer-2_S94_R2	17866582	13087586	73.25
JohnBrewer-3_S97_R1	15091104	12222232	80.99
JohnBrewer-3_S97_R2	15091104	12222232	80.99
JohnBrewer-4_S98_R1	18972654	15490727	81.65
JohnBrewer-4_S98_R2	18972654	15490727	81.65
Kelso-1_S85_R1	18183883	14573742	80.15
Kelso-1_S85_R2	18183883	14573742	80.15
Kelso-2_S86_R1	15760712	12396041	78.65
Kelso-2_S86_R2	15760712	12396041	78.65

Kelso-3_S87_R1	16438019	13053054	79.41
Kelso-3_S87_R2	16438019	13053054	79.41
Kelso-4_S88_R1	14210790	11115237	78.22
Kelso-4_S88_R2	14210790	11115237	78.22
Knife-1_S45_R1	10158905	7604403	74.85
Knife-1_S45_R2	10158905	7604403	74.85
Knife-2_S46_R1	10123508	7867437	77.71
Knife-2_S46_R2	10123508	7867437	77.71
Knife-3_S47_R1	13637941	10614685	77.83
Knife-3_S47_R2	13637941	10614685	77.83
Knife-4_S48_R1	12792248	9071031	70.91
Knife-4_S48_R2	12792248	9071031	70.91
Lagoon-1_S13_R1	23078370	18229727	78.99
Lagoon-1_S13_R2	23078370	18229727	78.99
Lagoon-2_S14_R1	19727931	15915193	80.67
Lagoon-2_S14_R2	19727931	15915193	80.67
Lagoon-3_S15_R1	20953079	17713070	84.54
Lagoon-3_S15_R2	20953079	17713070	84.54
Lagoon-4_S16_R1	22291127	17777877	79.75
Lagoon-4_S16_R2	22291127	17777877	79.75
LittleKelso-1_S81_R1	14780869	11862978	80.26
LittleKelso-1_S81_R2	14780869	11862978	80.26
LittleKelso-2_S82_R1	16355172	12732746	77.85
LittleKelso-2_S82_R2	16355172	12732746	77.85
LittleKelso-3_S83_R1	17771440	14193671	79.87
LittleKelso-3_S83_R2	17771440	14193671	79.87
LittleKelso-4_S84_R1	14134872	10782383	76.28
LittleKelso-4_S84_R2	14134872	10782383	76.28
Lynchs-1_S99_R1	17440400	13917717	79.8
Lynchs-1_S99_R2	17440400	13917717	79.8
Lynchs-2_S100_R1	13916361	10377547	74.57
Lynchs-2_S100_R2	13916361	10377547	74.57
Lynchs-3_S101_R1	18150036	14281813	78.69
Lynchs-3_S101_R2	18150036	14281813	78.69
Lynchs-4_S102_R1	16006063	12847768	80.27
Lynchs-4_S102_R2	16006063	12847768	80.27
Mantis-1_S85_R1	18993466	14558201	76.65
Mantis-1_S85_R2	18993466	14558201	76.65
Mantis-2_S86_R1	21619816	15635305	72.32
Mantis-2_S86_R2	21619816	15635305	72.32
Mantis-3_S87_R1	18180195	14425519	79.35
Mantis-3_S87_R2	18180195	14425519	79.35
Mantis-4_S88_R1	19588794	15831087	80.82
Mantis-4_S88_R2	19588794	15831087	80.82
Masthead-1_S53_R1	24458492	19381421	79.24
Masthead-1_S53_R2	24458492	19381421	79.24
Masthead-2_S54_R1	17157643	13676920	79.71
Masthead-2_S54_R2	17157643	13676920	79.71
Masthead-3_S55_R1	21267856	16708121	78.56
Masthead-3_S55_R2	21267856	16708121	78.56
Masthead-4_S56_R1	25186255	19727992	78.33
Masthead-4_S56_R2	25186255	19727992	78.33
McCulloch-1_S17_R1	16259643	13239204	81.42
McCulloch-1_S17_R2	16259643	13239204	81.42
McCulloch-2_S18_R1	15248329	12469021	81.77

McCulloch-2_S18_R2	15248329	12469021	81.77
McCulloch-3_S19_R1	21195708	17380964	82
McCulloch-3_S19_R2	21195708	17380964	82
McCulloch-4_S20_R1	12440154	10216023	82.12
McCulloch-4_S20_R2	12440154	10216023	82.12
McSweeney-1_S5_R1	21741600	17088561	78.6
McSweeney-1_S5_R2	21741600	17088561	78.6
McSweeney-2_S6_R1	19967227	15684275	78.55
McSweeney-2_S6_R2	19967227	15684275	78.55
McSweeney-3_S7_R1	27085493	22004592	81.24
McSweeney-3_S7_R2	27085493	22004592	81.24
McSweeney-4_S8_R1	23783727	18680695	78.54
McSweeney-4_S8_R2	23783727	18680695	78.54
Monsoon-1_S21_R1	20358282	14560732	71.52
Monsoon-1_S21_R2	20358282	14560732	71.52
Monsoon-2_S22_R1	23634715	18519565	78.36
Monsoon-2_S22_R2	23634715	18519565	78.36
Monsoon-3_S23_R1	19236392	15353922	79.82
Monsoon-3_S23_R2	19236392	15353922	79.82
Monsoon-4_S24_R1	20673438	16903268	81.76
Monsoon-4_S24_R2	20673438	16903268	81.76
Moore-1_S25_R1	18190924	14597050	80.24
Moore-1_S25_R2	18190924	14597050	80.24
Moore-2_S26_R1	14319030	11477735	80.16
Moore-2_S26_R2	14319030	11477735	80.16
Moore-3_S27_R1	12845546	10118946	78.77
Moore-3_S27_R2	12845546	10118946	78.77
Moore-4_S28_R1	15775687	12604975	79.9
Moore-4_S28_R2	15775687	12604975	79.9
Myrmidon-1_S53_R1	9585650	7487447	78.11
Myrmidon-1_S53_R2	9585650	7487447	78.11
Myrmidon-2_S54_R1	11445140	8866528	77.47
Myrmidon-2_S54_R2	11445140	8866528	77.47
Myrmidon-3_S55_R1	11835347	9243782	78.1
Myrmidon-3_S55_R2	11835347	9243782	78.1
Myrmidon-4_S56_R1	12884881	10408827	80.78
Myrmidon-4_S56_R2	12884881	10408827	80.78
North-1_S37_R1	15893048	12712728	79.99
North-1_S37_R2	15893048	12712728	79.99
North-2_S38_R1	19790656	16189274	81.8
North-2_S38_R2	19790656	16189274	81.8
North-3_S39_R1	24246873	19981797	82.41
North-3_S39_R2	24246873	19981797	82.41
North-4_S40_R1	19528038	16037916	82.13
North-4_S40_R2	19528038	16037916	82.13
Peart-1_S13_R1	12498052	10061259	80.5
Peart-1_S13_R2	12498052	10061259	80.5
Peart-2_S14_R1	13788722	11231822	81.46
Peart-2_S14_R2	13788722	11231822	81.46
Peart-3_S15_R1	11397354	9065275	79.54
Peart-3_S15_R2	11397354	9065275	79.54
Peart-4_S16_R1	12814561	9894545	77.21
Peart-4_S16_R2	12814561	9894545	77.21
Rib-1_S73_R1	16349857	12907883	78.95
Rib-1_S73_R2	16349857	12907883	78.95

Rib-2_S74_R1	18617365	14801568	79.5
Rib-2_S74_R2	18617365	14801568	79.5
Rib-3_S75_R1	19502285	15618797	80.09
Rib-3_S75_R2	19502285	15618797	80.09
Rib-4_S76_R1	17561081	13809062	78.63
Rib-4_S76_R2	17561081	13809062	78.63
Roxburgh-1_S89_R1	15711737	12658785	80.57
Roxburgh-1_S89_R2	15711737	12658785	80.57
Roxburgh-2_S90_R1	17574836	13869737	78.92
Roxburgh-2_S90_R2	17574836	13869737	78.92
Roxburgh-3_S91_R1	14481635	11658659	80.51
Roxburgh-3_S91_R2	14481635	11658659	80.51
Roxburgh-4_S92_R1	16622610	13266811	79.81
Roxburgh-4_S92_R2	16622610	13266811	79.81
Sanbank1-1_S77_R1	21458501	17368212	80.94
Sanbank1-1_S77_R2	21458501	17368212	80.94
Sanbank1-2_S78_R1	18263182	15192787	83.19
Sanbank1-2_S78_R2	18263182	15192787	83.19
Sanbank1-3_S79_R1	23266034	18571860	79.82
Sanbank1-3_S79_R2	23266034	18571860	79.82
Sanbank1-4_S80_R1	19199687	15386064	80.14
Sanbank1-4_S80_R2	19199687	15386064	80.14
SmallLagoon-1_S45_R1	20241764	16190538	79.99
SmallLagoon-1_S45_R2	20241764	16190538	79.99
SmallLagoon-2_S46_R1	22897812	19044958	83.17
SmallLagoon-2_S46_R2	22897812	19044958	83.17
SmallLagoon-3_S47_R1	19426244	15977442	82.25
SmallLagoon-3_S47_R2	19426244	15977442	82.25
SmallLagoon-4_S48_R1	16892626	13129683	77.72
SmallLagoon-4_S48_R2	16892626	13129683	77.72
St-Crispin-1_S73_R1	20269787	16730735	82.54
St-Crispin-1_S73_R2	20269787	16730735	82.54
St-Crispin-2_S74_R1	22821633	18780890	82.29
St-Crispin-2_S74_R2	22821633	18780890	82.29
St-Crispin-3_S75_R1	22609122	17726032	78.4
St-Crispin-3_S75_R2	22609122	17726032	78.4
St-Crispin-4_S76_R1	15583537	12224682	78.45
St-Crispin-4_S76_R2	15583537	12224682	78.45
Taylor-1_S9_R1	19551092	15557593	79.57
Taylor-1_S9_R2	19551092	15557593	79.57
Taylor-2_S10_R1	13338249	10343864	77.55
Taylor-2_S10_R2	13338249	10343864	77.55
Taylor-3_S11_R1	14349624	11147360	77.68
Taylor-3_S11_R2	14349624	11147360	77.68
Taylor-4_S12_R1	11147073	8838527	79.29
Taylor-4_S12_R2	11147073	8838527	79.29
Thetford-1_S29_R1	19374041	16034796	82.76
Thetford-1_S29_R2	19374041	16034796	82.76
Thetford-2_S30_R1	16355562	12740151	77.89
Thetford-2_S30_R2	16355562	12740151	77.89
Thetford-3_S31_R1	18511046	15217895	82.21
Thetford-3_S31_R2	18511046	15217895	82.21
Thetford-4_S32_R1	15617475	12342274	79.03
Thetford-4_S32_R2	15617475	12342274	79.03

**SUM**	6566753254	5209101390	
**AVERAGE**	17464769	13853993	78.84
**SD**	4075365	3324976	

Table S2. Final Illumina sequencing counts after 5 additional filtering steps in R, i.e. after removing (1) non-annotated reads; taxa annotated as (2) eukaryotic or (3) viral; (4) prokaryotic reads annotated to the Domain level only (Bacteria or Archaea); and (5) rare/spurious reads (relative abundance < 0.0001%). These values are only reported for Forward reads (R1 samples).

Sample_ID	Final seq counts after filtering steps in R
11-049-1_S89_R1	2238268
11-049-2_S90_R1	1860457
11-049-3_S91_R1	3833908
11-049-4_S92_R1	4172951
11-162-1_S81_R1	2557283
11-162-2_S82_R1	2422365
11-162-3_S83_R1	3250101
11-162-4_S84_R1	3576607
13-124-1_S9_R1	5618759
13-124-2_S10_R1	4042401
13-124-3_S11_R1	3902277
13-124-4_S12_R1	5445228
21-550-1_S69_R1	3933607
21-550-2_S70_R1	5813631
21-550-3_S71_R1	4201402
21-550-4_S72_R1	5071162
21-580-1_S57_R1	5473576
21-580-2_S58_R1	2655361
21-580-3_S59_R1	4814692
21-580-4_S60_R1	4332709
22-084-1_S41_R1	3697802
22-084-2_S42_R1	1552185
22-084-3_S43_R1	3827890
22-084-4_S44_R1	1935327
Agincourt1-1_S33_R1	3182762
Agincourt1-2_S34_R1	3883212
Agincourt1-3_S35_R1	5942033
Agincourt1-4_S36_R1	7261123
Arlington-1_S37_R1	2671719
Arlington-2_S38_R1	2434265
Arlington-3_S39_R1	2077177
Arlington-4_S40_R1	3559374
Boult-1_S25_R1	2637146
Boult-2_S26_R1	5374206
Boult-3_S27_R1	2011943
Boult-4_S28_R1	3685480
Broomfield-1_S49_R1	4239082
Broomfield-3_S51_R1	4188500
Broomfield-4_S52_R1	6553403
Broomfield-rpt-2_S115_R1	2847907
Centipede-1_S57_R1	2487830
Centipede-2_S58_R1	2239725

Centipede-3_S59_R1	2089218
Centipede-4_S60_R1	1930925
Chicken-1_S69_R1	2743241
Chicken-2_S70_R1	2294721
Chicken-3_S71_R1	2348571
Chicken-4_S72_R1	3456855
Chinaman-1_S65_R1	6186135
Chinaman-2_S66_R1	3554043
Chinaman-3_S67_R1	3681207
Chinaman-4_S68_R1	3478148
Corbett-1_S17_R1	3139340
Corbett-2_S18_R1	4664409
Corbett-3_S19_R1	4613079
Corbett-4_S20_R1	4215218
Davie-1_S1_R1	4507298
Davie-2_S2_R1	3824003
Davie-3_S3_R1	4064695
Davie-4_S4_R1	3023787
Erskine-1_S61_R1	3225637
Erskine-2_S62_R1	3109453
Erskine-3_S63_R1	3534047
Erskine-4_S64_R1	4449051
Fairfax-1_S33_R1	4289235
Fairfax-2_S34_R1	5064650
Fairfax-3_S35_R1	4434434
Fairfax-4_S36_R1	4869504
Farquaharson-1_S1_R1	2997655
Farquaharson-2_S2_R1	2546533
Farquaharson-3_S3_R1	2392144
Farquaharson-4_S4_R1	2566055
Feather-1_S5_R1	2109148
Feather-2_S6_R1	3244705
Feather-3_S7_R1	3206451
Feather-4_S8_R1	2121799
Fore-and-Aft-1_S77_R1	2659220
Fore-and-Aft-2_S78_R1	2741565
Fore-and-Aft-3_S79_R1	3111442
Fore-and-Aft-4_S80_R1	3126931
Fork-1_S49_R1	3121550
Fork-2_S50_R1	3359527
Fork-3_S51_R1	2749999
Fork-4_S52_R1	2611775
Grub-1_S65_R1	3512104
Grub-2_S66_R1	2747673
Grub-3_S67_R1	3594722
Grub-4_S68_R1	3092440
Hastings-1_S41_R1	2844155
Hastings-2_S42_R1	2700457
Hastings-3_S43_R1	4246952
Hastings-4_S44_R1	4142067
Hedley-1_S21_R1	4468131
Hedley-2_S22_R1	3538499
Hedley-3_S23_R1	4084295
Helix-1_S61_R1	1416015
Helix-2_S62_R1	2985280

Helix-3_S63_R1	2249809
Helix-4_S64_R1	3076135
Hoskyn-1_S29_R1	5478099
Hoskyn-2_S30_R1	6564116
Hoskyn-3_S31_R1	6441169
Hoskyn-4_S32_R1	4973817
JohnBrewer-1_S93_R1	2374532
JohnBrewer-2_S94_R1	2767402
JohnBrewer-3_S97_R1	2790928
JohnBrewer-4_S98_R1	2384057
Kelso-1_S85_R1	1965242
Kelso-2_S86_R1	2944436
Kelso-3_S87_R1	3023374
Kelso-4_S88_R1	2403779
Knife-1_S45_R1	2169265
Knife-2_S46_R1	2127048
Knife-3_S47_R1	2651727
Knife-4_S48_R1	2326374
Lagoon-1_S13_R1	7060303
Lagoon-2_S14_R1	6878468
Lagoon-3_S15_R1	7063410
Lagoon-4_S16_R1	4659824
LittleKelso-1_S81_R1	3125022
LittleKelso-2_S82_R1	4043485
LittleKelso-3_S83_R1	3250841
LittleKelso-4_S84_R1	2859866
Lynchs-1_S99_R1	1178646
Lynchs-2_S100_R1	1975117
Lynchs-3_S101_R1	1827584
Lynchs-4_S102_R1	1231772
Mantis-1_S85_R1	4695408
Mantis-2_S86_R1	5249588
Mantis-3_S87_R1	4650009
Mantis-4_S88_R1	2971803
Masthead-1_S53_R1	8407104
Masthead-2_S54_R1	5536049
Masthead-3_S55_R1	6379221
Masthead-4_S56_R1	7638936
McCulloch-1_S17_R1	3655307
McCulloch-2_S18_R1	2475985
McCulloch-3_S19_R1	4156655
McCulloch-4_S20_R1	2423251
McSweeney-1_S5_R1	4032218
McSweeney-2_S6_R1	3414588
McSweeney-3_S7_R1	6295922
McSweeney-4_S8_R1	6238959
Monsoon-1_S21_R1	3667650
Monsoon-2_S22_R1	6003637
Monsoon-3_S23_R1	4099860
Monsoon-4_S24_R1	6054502
Moore-1_S25_R1	6014109
Moore-2_S26_R1	3809722
Moore-3_S27_R1	3603913
Moore-4_S28_R1	3313231
Myrmidon-1_S53_R1	2379442

Myrmidon-2_S54_R1	2885702
Myrmidon-3_S55_R1	3070913
Myrmidon-4_S56_R1	3465757
North-1_S37_R1	4031656
North-2_S38_R1	5750934
North-3_S39_R1	7688579
North-4_S40_R1	4510850
Peart-1_S13_R1	2708995
Peart-2_S14_R1	2910381
Peart-3_S15_R1	2243220
Peart-4_S16_R1	2686266
Rib-1_S73_R1	2825536
Rib-2_S74_R1	3876646
Rib-3_S75_R1	3750262
Rib-4_S76_R1	3607788
Roxburgh-1_S89_R1	3088907
Roxburgh-2_S90_R1	3573274
Roxburgh-3_S91_R1	2739937
Roxburgh-4_S92_R1	3218792
Sanbank1-1_S77_R1	5400936
Sanbank1-2_S78_R1	5463260
Sanbank1-3_S79_R1	5169186
Sanbank1-4_S80_R1	4884874
SmallLagoon-1_S45_R1	5114622
SmallLagoon-2_S46_R1	5604996
SmallLagoon-3_S47_R1	5337066
SmallLagoon-4_S48_R1	2878122
St-Crispin-1_S73_R1	4035898
St-Crispin-2_S74_R1	4207828
St-Crispin-3_S75_R1	5144414
St-Crispin-4_S76_R1	3645959
Taylor-1_S9_R1	4002316
Taylor-2_S10_R1	2505286
Taylor-3_S11_R1	2768573
Taylor-4_S12_R1	2292486
Thetford-1_S29_R1	5646577
Thetford-2_S30_R1	3333830
Thetford-3_S31_R1	4591474
Thetford-4_S32_R1	2639751
**Average**	3752207
**SD**	1402667

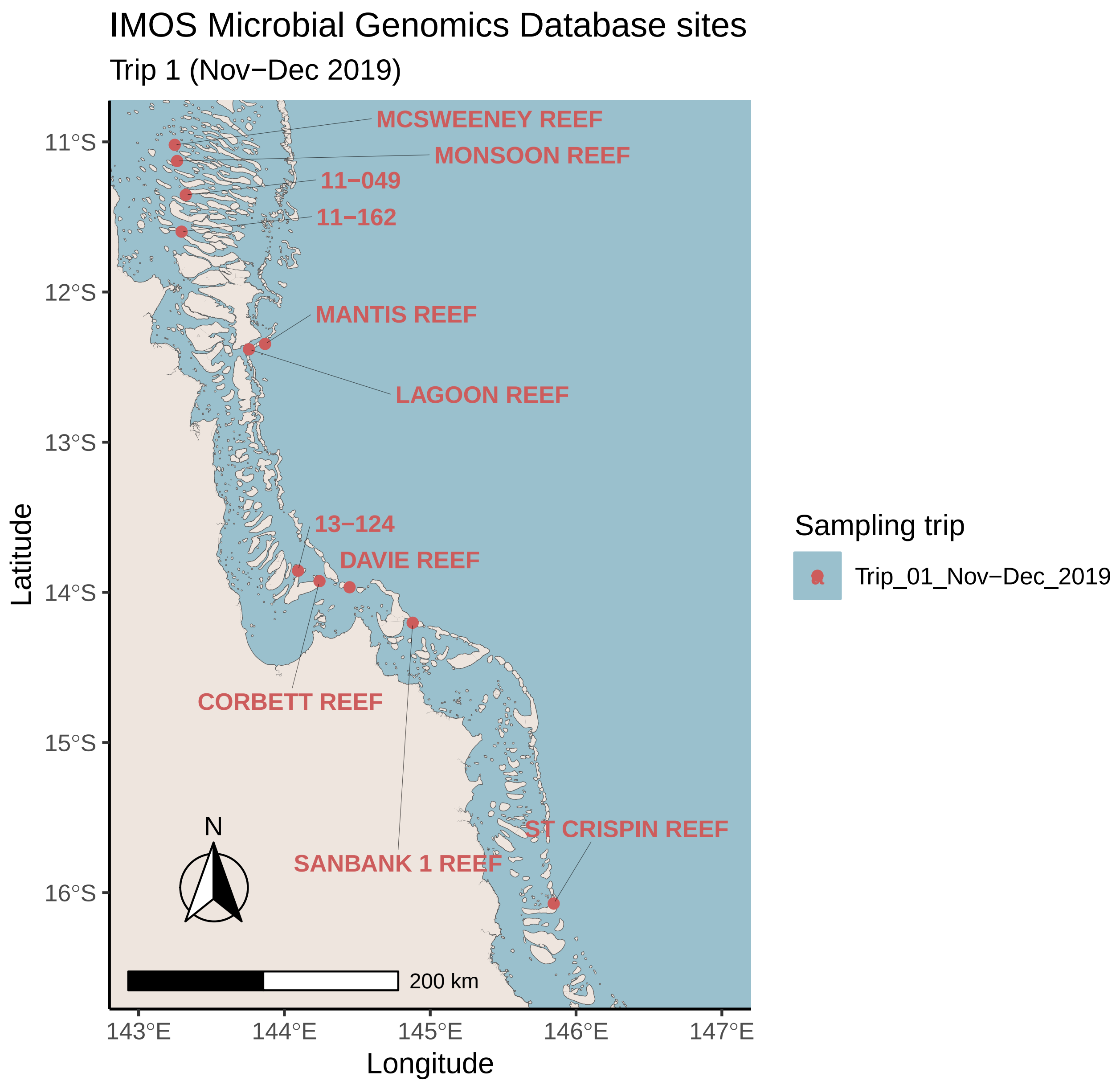

Figure S1: Reef sites pertinent to the Trip 1 sampling event, conducted in November and December 2019, in the Cape Grenville and Princess Charlotte bay sectors of the northern GBR.

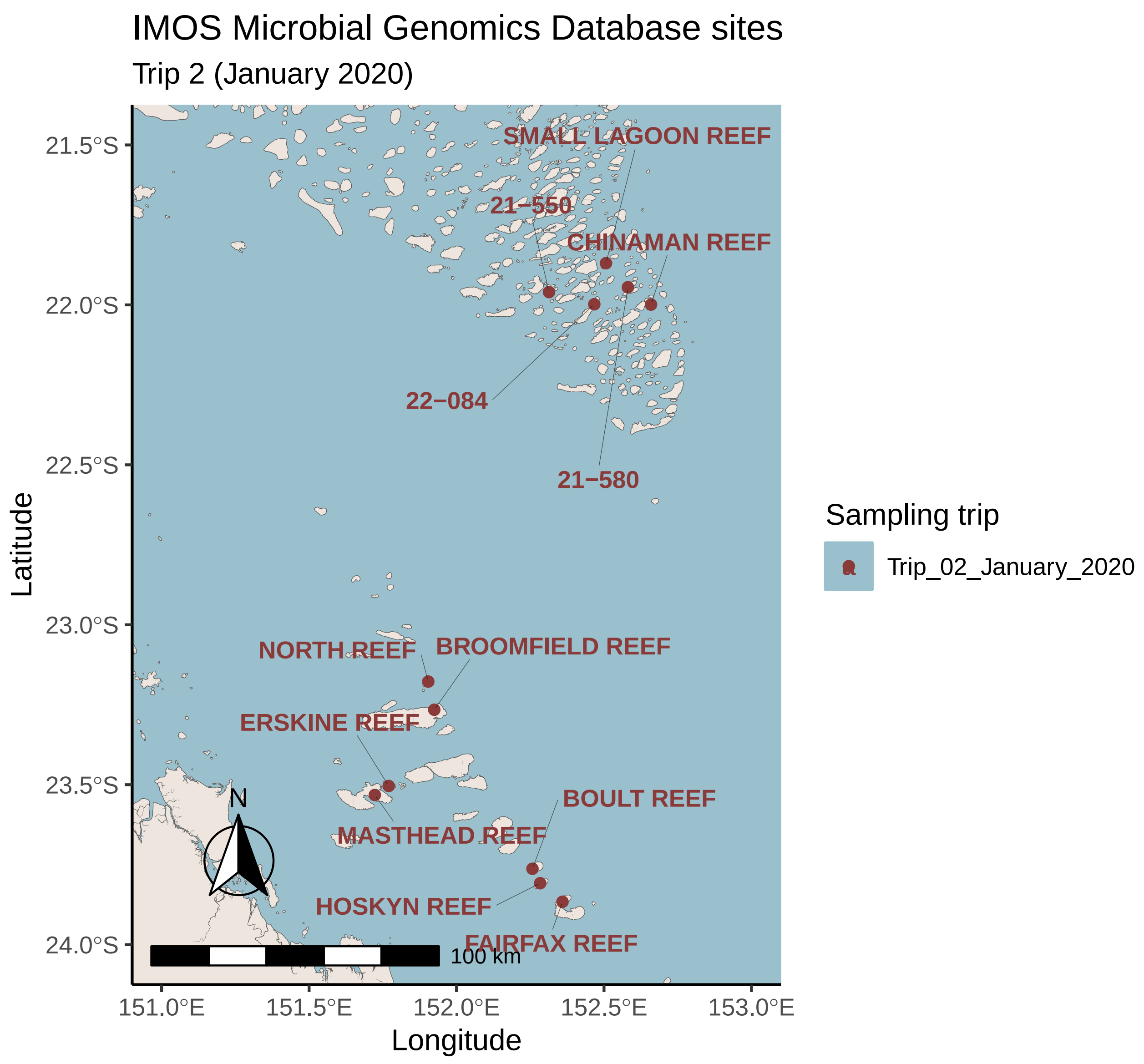

Figure S2: Reef sites pertinent to the Trip 2 sampling event, conducted in January 2020, in the Swains and Capricorn Bunker sectors of the southern GBR.

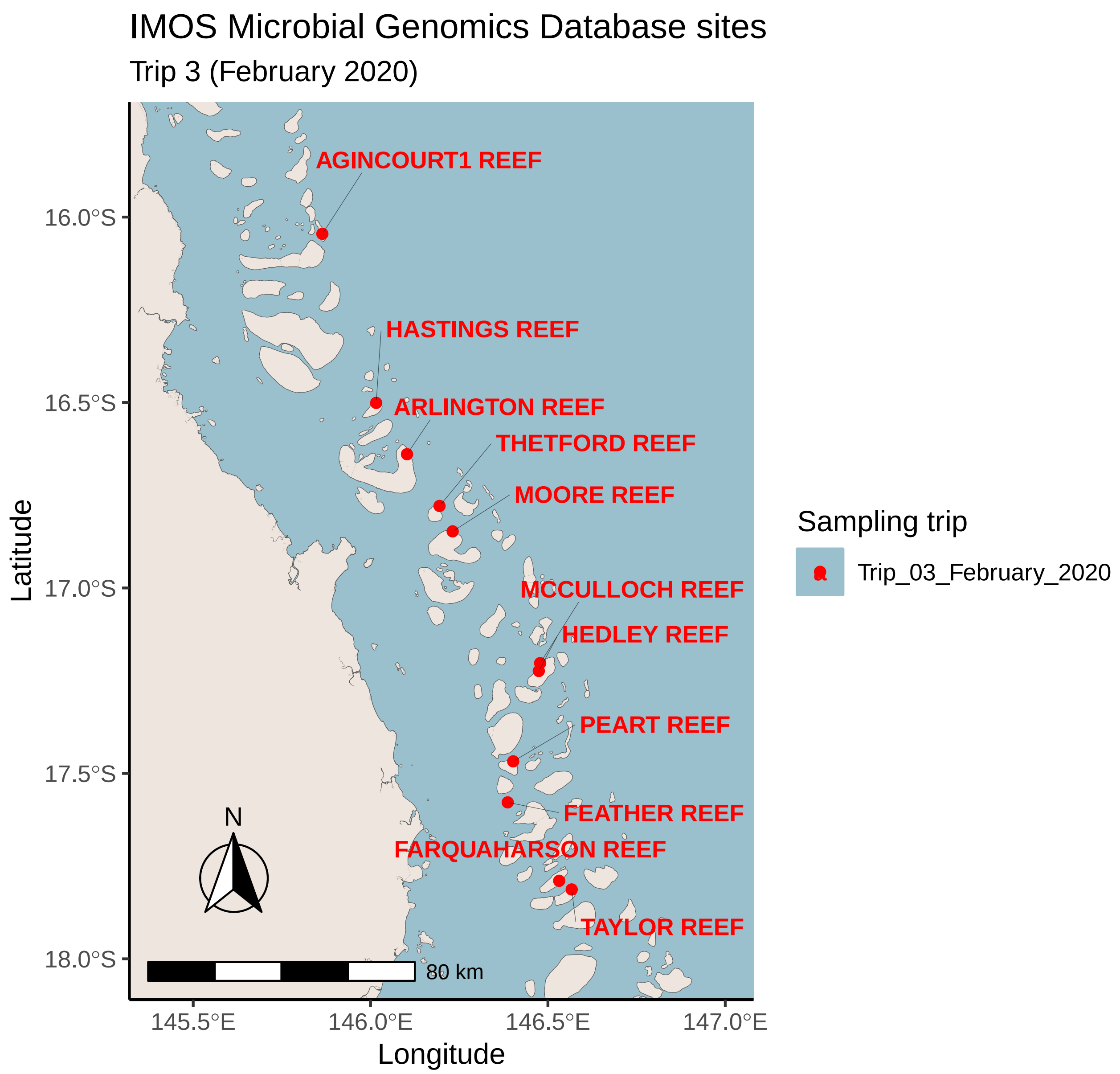

Figure S3: Reef sites pertinent to the Trip 3 sampling event, conducted in February 2020, in the Cairns and Inninsfail sectors of the central GBR.

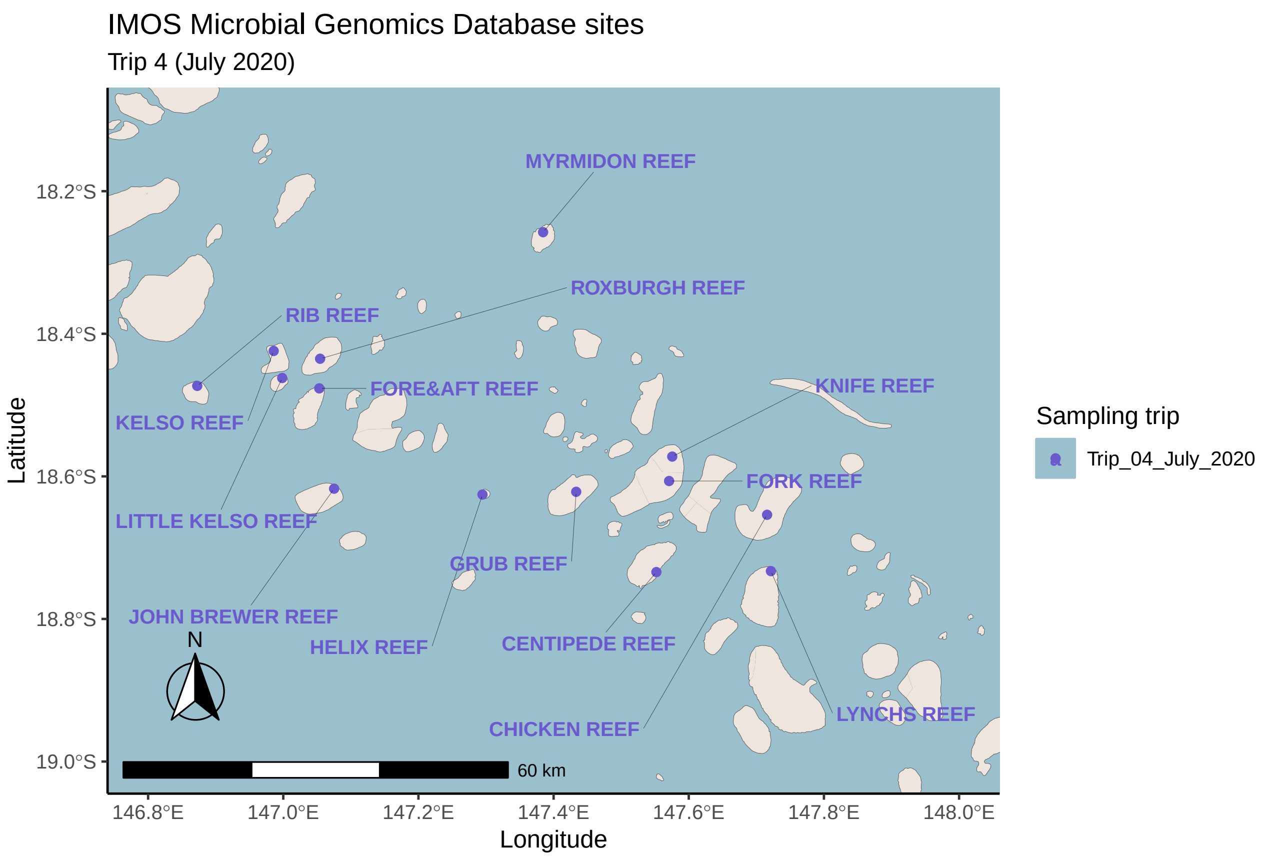

Figure S4: Reef sites pertinent to the Trip 4 sampling event, conducted in July 2020, in the Townsville sector of the central GBR.

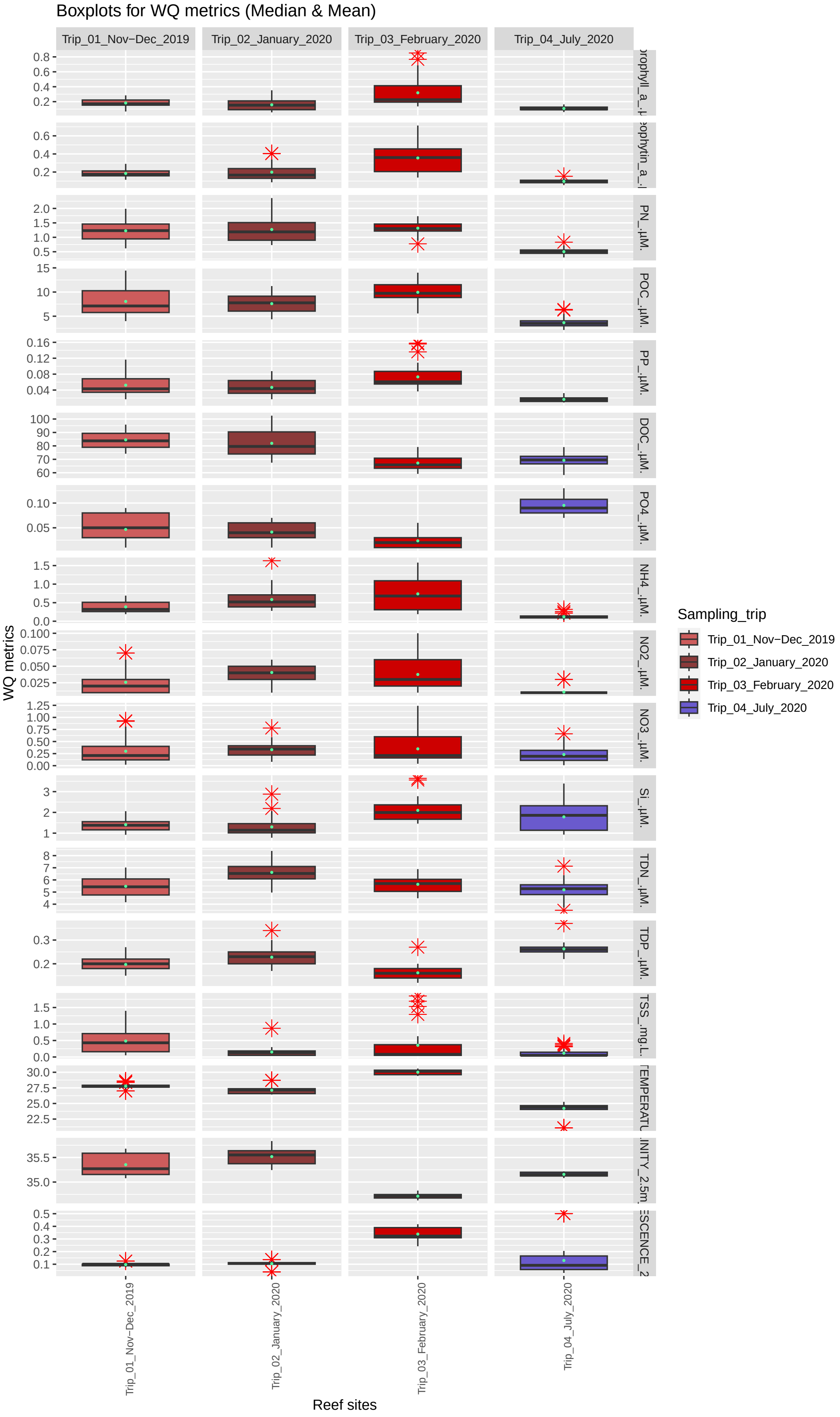

Figure S5: Physico-chemical data. Median ± SD values of 17 physico-chemical variables collected. Values are summarised across the four sampling trips, with the colour code corresponding to Fig. 1 in the main text.

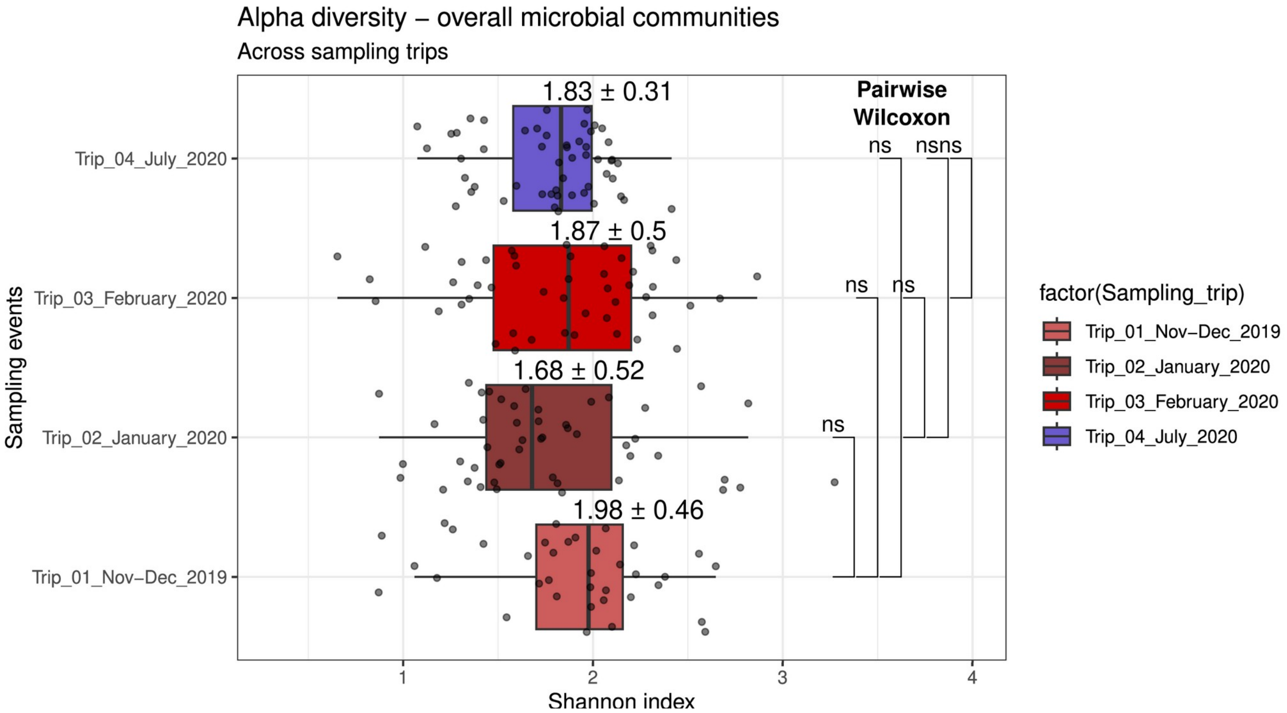

Figure S6. Boxplots illustrate microbial diversity (Shannon Index) for genera *for overall microbial communities*, across sampling trips. The symbols *, **, ***, and **** denote levels of statistical significance in pairwise Wilcoxon rank sum tests when testing variation of Shannon diversity scores for overall microbial communities across the four sampling trips: * for $p < 0.05$; ** for $p < 0.01$; *** for $p < 0.001$; and **** for $p < 0.0001$, indicating increasing levels of significance. 'ns' indicates non-significant results, where $p \geq 0.05$.

Table S3. Median and standard deviation for Shannon Index values, computed within trips.

Sampling_trip	Median	SD
Trip_01_Nov-Dec_2019	1.976648	0.4646255
Trip_02_January_2020	1.678609	0.5192536
Trip_03_February_2020	1.871546	0.4974857
Trip_04_July_2020	1.831793	0.3093137

Table S4. Pairwise Wilcoxon rank sum tests to compare median Shannon Diversity between sampling trips, computed for overall communities.

group1	group2	n1	n2	statistic	p	p.adj	p.adj.signif
Trip_01_Nov-Dec_2019	Trip_02_January_2020	36	48	1030	0.135	0.81	ns
Trip_01_Nov-Dec_2019	Trip_03_February_2020	36	47	887	0.711	1.00	ns
Trip_01_Nov-Dec_2019	Trip_04_July_2020	36	52	1106	0.150	0.81	ns
Trip_02_January_2020	Trip_03_February_2020	48	47	1010	0.383	1.00	ns
Trip_02_January_2020	Trip_04_July_2020	48	52	1139	0.454	1.00	ns
Trip_03_February_2020	Trip_04_July_2020	47	52	1361	0.332	1.00	ns

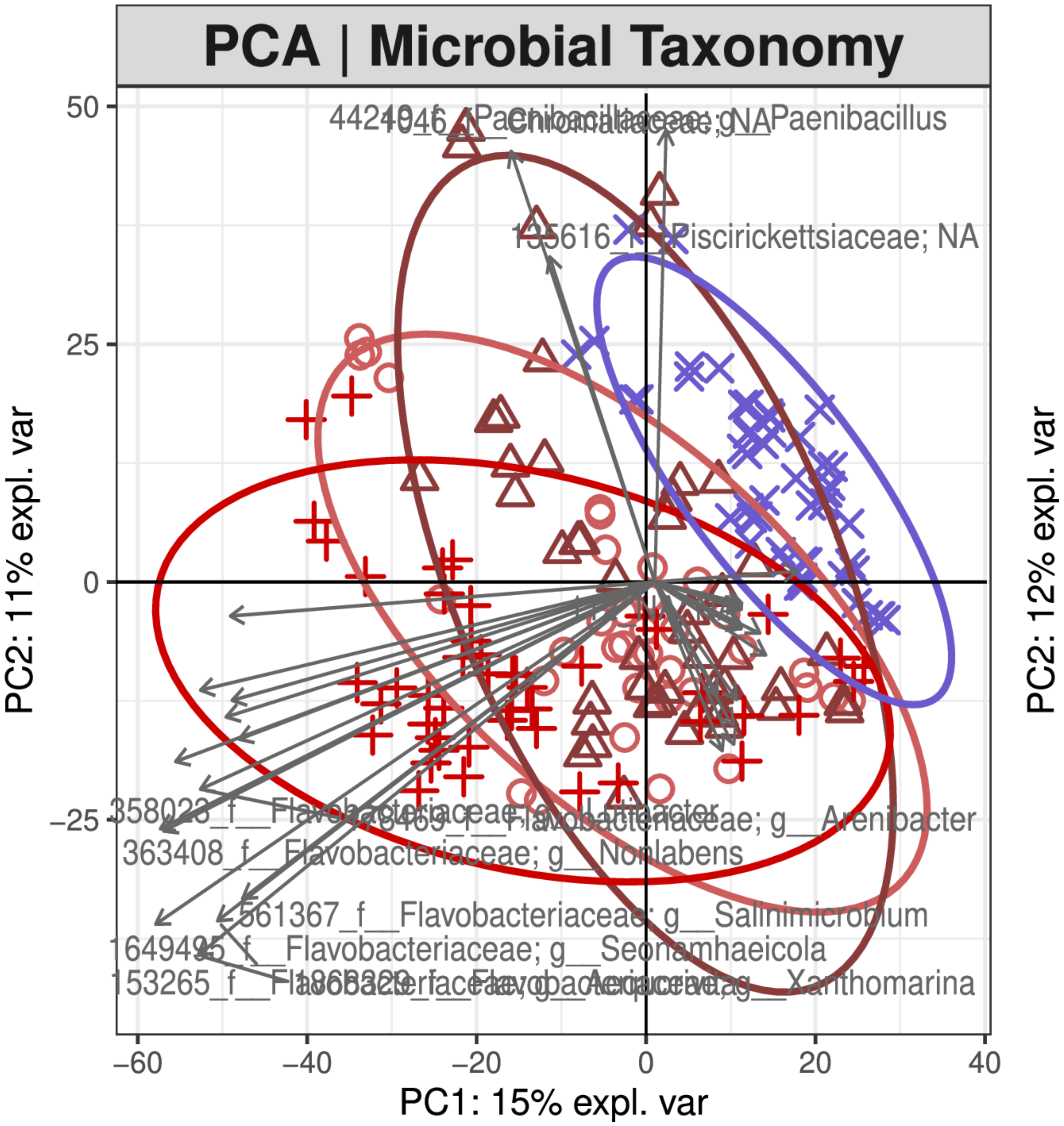

Figure S7: Biplots from the Principal Components Analysis (PCA) show the main clustering patterns of reef sites based on microbial taxonomic community composition. The plots highlight which microbial taxa are enriched in specific reef sites, coloured in red or blue tones to denote trips that occurred during the austral summer (wet season) or austral winter (dry season), respectively.

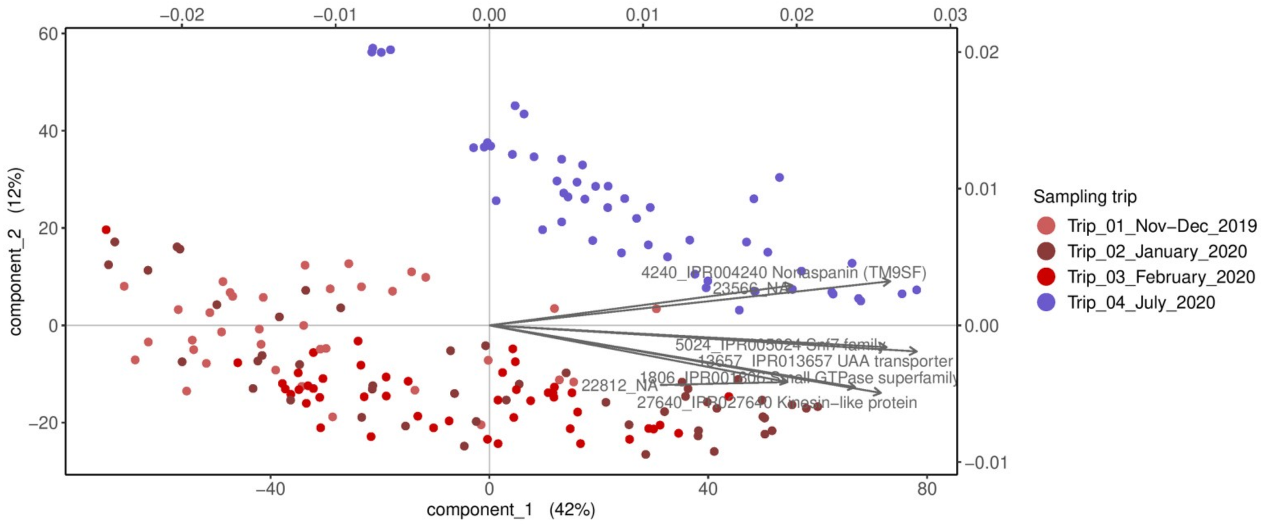

Figure S8: Biplots from the Principal Components Analysis (PCA) show the main clustering patterns of reef sites based on microbial functional community composition (i.e. GO terms). The plots highlight which microbial genes are enriched in specific reef sites, coloured in red or blue tones to denote trips that occurred during the austral summer (wet season) or austral winter (dry season), respectively.

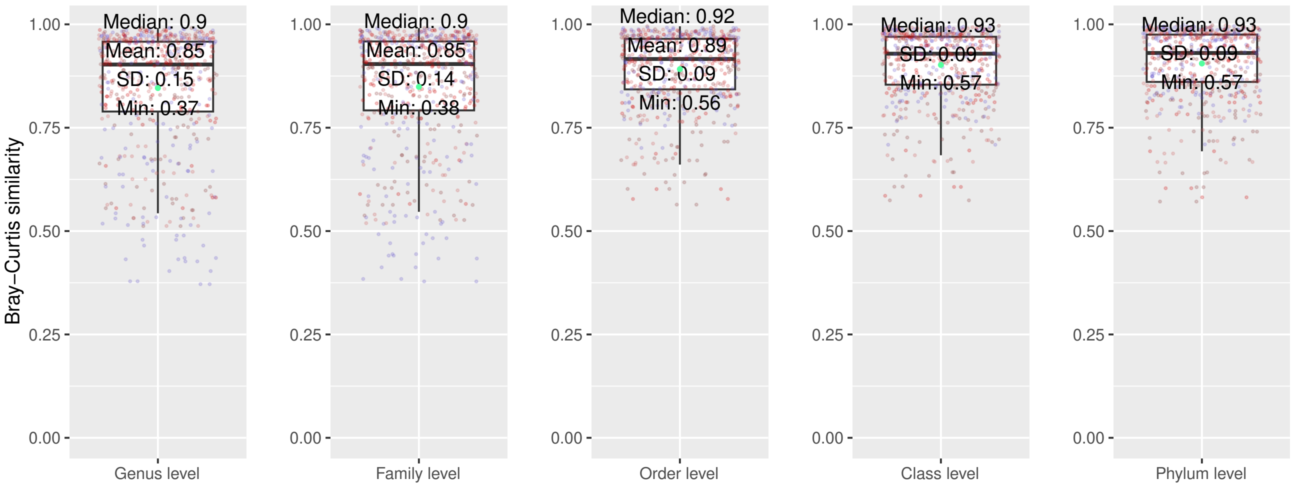

Figure S9: Bray-Curtis Similarity Index shows within-reef community similarity (0 = dissimilar; 1 = identical) for microbial taxonomy at genus, family, order, class, and phylum-level classifications. Data points are coloured-coded to correspond the colouring scheme in Fig. 1 in the main text, and the green dot represents the mean Bray-Curtis similarity.
